# PIF4 enhances DNA binding of CDF2 to co-regulate target gene expression and promote Arabidopsis hypocotyl cell elongation

**DOI:** 10.1038/s41477-022-01213-y

**Published:** 2022-08-15

**Authors:** He Gao, Wen Song, Edouard Severing, Alice Vayssières, Bruno Huettel, Rainer Franzen, René Richter, Jijie Chai, George Coupland

**Affiliations:** 1 Department of Plant Developmental Biology, Max Planck Institute for Plant Breeding Research, Cologne, Germany; 2 Institute of Biochemistry, University of Cologne, Cologne, Germany

**Keywords:** Plant molecular biology, Plant morphogenesis, Light responses

## Abstract

How specificity is conferred within gene regulatory networks is an important problem in biology. The basic helix–loop–helix PHYTOCHROME-INTERACTING FACTORs (PIFs) and single zinc-finger CYCLING DOF FACTORs (CDFs) mediate growth responses of Arabidopsis to light and temperature. We show that these two classes of transcription factor (TF) act cooperatively. CDF2 and PIF4 are temporally and spatially co-expressed, they interact to form a protein complex and act in the same genetic pathway to promote hypocotyl cell elongation. Furthermore, PIF4 substantially strengthens genome-wide occupancy of CDF2 at a subset of its target genes. One of these, *YUCCA8*, encodes an auxin biosynthesis enzyme whose transcription is increased by PIF4 and CDF2 to contribute to hypocotyl elongation. The binding sites of PIF4 and CDF2 in *YUCCA8* are closely spaced, and in vitro PIF4 enhances binding of CDF2. We propose that this occurs by direct protein interaction and because PIF4 binding alters DNA conformation. Thus, we define mechanisms by which PIF and CDF TFs cooperate to achieve regulatory specificity and promote cell elongation in response to light.

## Abstract (web summary)

Hypocotyl cell elongation is promoted by combinatorial action of PIF4 and CDF2. This occurs because PIF4 enhances binding of CDF2 to common target genes increasing their transcription. These genes include the auxin biosynthesis gene *YUCCA8*.

## Main

Cellular responses to environmental and developmental signals require activation of gene regulatory networks by recruitment of transcription factors (TFs) to specific genes^1^. Plant genomes encode relatively large numbers of TFs^2^, emphasizing the importance of transcriptional regulation, and recruitment of combinations of TFs to the same gene can integrate different signals and enhance specificity^3–5^. Nevertheless, TFs usually recognize simple DNA sequences in vitro, and it remains unclear how they are recruited to specific genes and implement unique functions in vivo. In plants, DOF (DNA-binding with one finger) TFs, which contain a conserved CX_2_CX_21_CX_2_C motif, regulate a wide range of developmental and environmental responses by binding to specific target genes in vivo^6^, but their DNA-binding site has only been described by a simple consensus motif, AAAG or [T/A]AAAG^7,8^, which occurs very widely in plant genomes. Within this family, CYCLING DOF FACTORS (CDF) are temporally regulated by the circadian clock to repress photoperiodic flowering and tuberisation^9–11^, and they also promote hypocotyl elongation and regulate abiotic stress responses^12–14^, but how they are recruited to specific target genes is unknown.

Combinatorial action of TFs can confer specificity in vivo and multiprotein TF complexes can exhibit new recognition properties and enhanced specificity for selected genes in vivo^15^. Some DOF proteins interact with other TFs or proteins to regulate gene transcription^6^, but it remains unclear how these interactions influence binding-site selection. Here we use a combination of in vivo and in vitro approaches to show that in Arabidopsis, CDF2 physically interacts with PHYTOCHROME-INTERACTING 4 (PIF4), an intensively studied basic helix–loop–helix (bHLH) TF with well-established functions in promoting growth in response to light and temperature^16–20^. PIF4 interacts directly with phytochrome and cryptochrome photoreceptors^18,19,21,22^, which regulate its activity in response to red/far-red and blue light, respectively. We find that PIF4 and CDF2 promote hypocotyl cell elongation, that the proteins directly interact and that PIF4 binding increases the strength and alters the specificity of CDF2 binding to a subset of target genes in vivo and in vitro. Therefore, combinatorial functions of PIF4 and CDF2 increase transcription of their mutual target genes, and provide a mechanism by which PIF4 enables CDF2 to activate specific target genes to promote hypocotyl cell growth.

## Results

### PIF4 and CDFs promote elongation of hypocotyl cells

**Extended Data Fig. 1:**
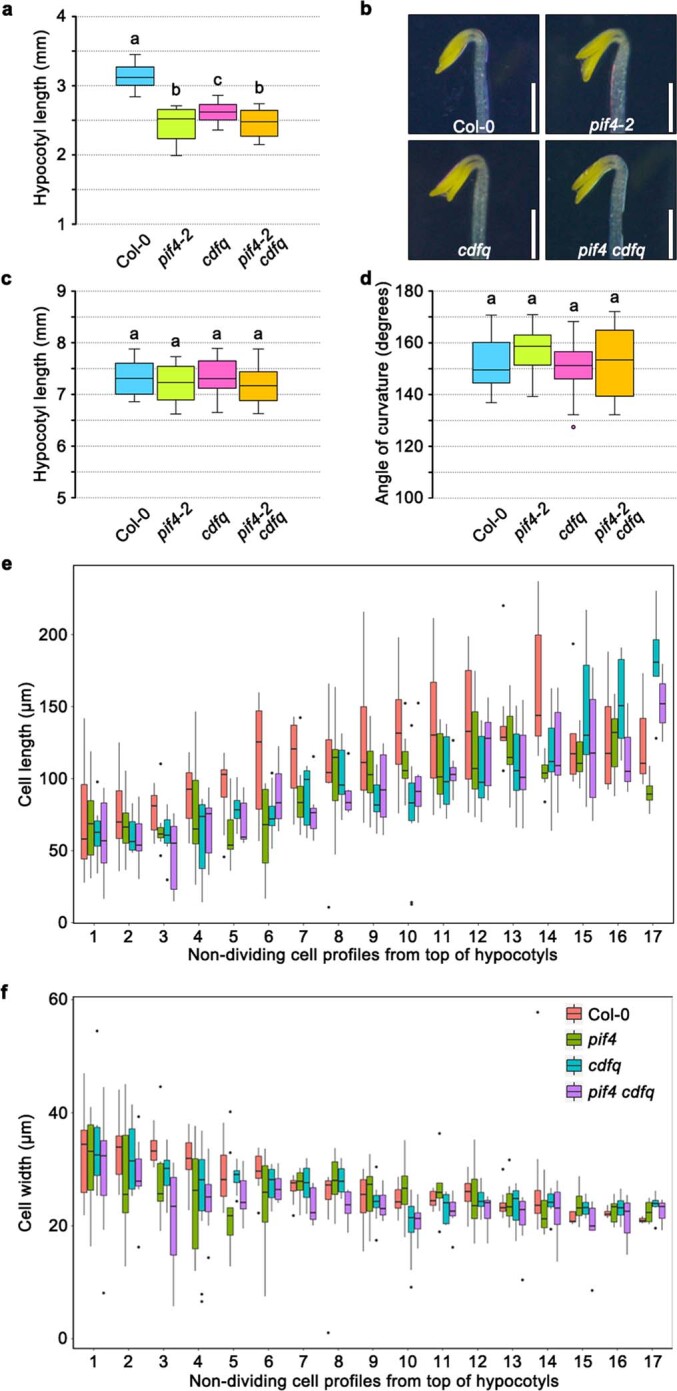
CDF1, 2, 3 and 5 function in the same genetic pathway with PIF4 to induce hypocotyl elongation. CDF1, 2, 3 and 5 function in the same genetic pathway with PIF4 to induce hypocotyl elongation. **a**, Hypocotyl length of 9-day-old seedlings of wild type, *pif4*, *cdfq* and *pif4 cdfq* in short-day conditions. **b**, Photographs of 4-day-old dark-grown seedlings. Scale bar = 1 mm. **c**, Hypocotyl length and **d**, hook curvature of 4-day-old dark-grown seedlings of WT, *pif4*, *cdfq* and *pif4 cdfq*. **e**, Length and **f**, width of cells in the non-dividing cell files of hypocotyls. Box plots in panel **a**, **b**, **d**, **e** and **f** show the minimum, 25^th^ percentile, median, 75^th^ percentile and maximum of data points. In **a** and **c**, *n* = 29 and 28 hypocotyls. *n* = 37 seedling hooks in **d** and *n* = 8 cell files examined over four hypocotyls (cell numbers are presented in Fig. 1b) in **e** and **f**. Letters a–c in panels **a**, **b** and **d** show significant differences between genotypes (*P* < 0.05, using one-way ANOVA followed by Tukey test, two sided), *P* = 1.96*10^−28^ in **a**, *P* = 0.16 in **b** and *P* = 0.067 in **d**. Source data

Under short-day (SD) photoperiods, the hypocotyl of Arabidopsis seedlings grows rhythmically with a peak in growth rate at dawn^23,24^. CDF and PIF TFs promote hypocotyl elongation under SDs^23,25,26^. Inactivation of the partially redundant *CDF1*, *CDF2*, *CDF3* and *CDF5* genes in the *cdf1235* quadruple (*cdfq*) mutant or of the *PIF4* and *PIF5* genes in the *pif4 pif5* double mutant reduces hypocotyl growth under SDs^14,23,25,26^. To test whether CDFs and PIF4 promote growth in the same genetic pathway, the *cdfq pif4* quintuple mutant was generated. In SDs, no differences were observed in hypocotyl length among the *pif4*, *cdfq* and *cdfq pif4* genotypes, although they all produced shorter hypocotyls than those of wild-type (Col-0) plants (Extended Data Fig. 1a). After germination in the dark, the length of the hypocotyl of *pif4*, *cdfq* and *cdfq pif4* mutants was indistinguishable from that of Col-0 (Extended Data Fig. 1b–d). Therefore, CDFs and PIF4 promote hypocotyl elongation under SDs in a non-additive, light-dependent manner, suggesting that they promote growth in the same genetic pathway.

**Fig. 1:**
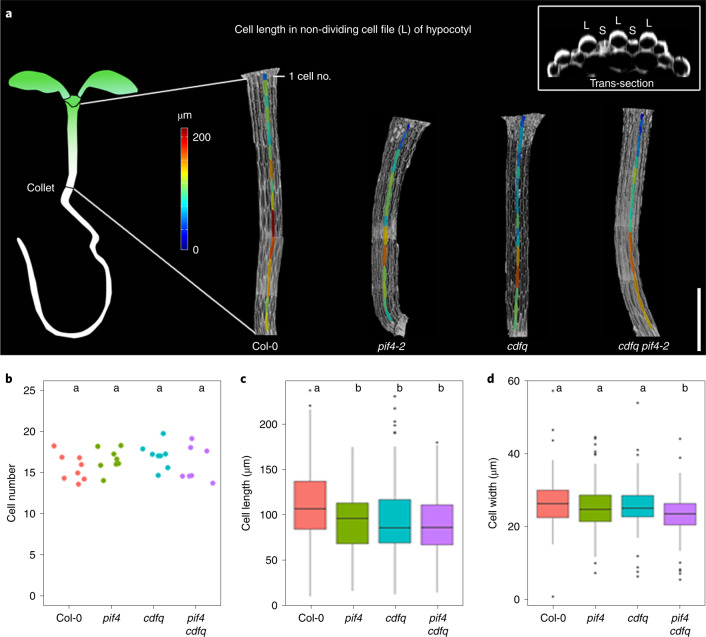
Analysis of epidermal cells in non-dividing cell files of Arabidopsis hypocotyls. **a**, Heatmap quantification of cell length in the non-dividing cell files of Col-0, *pif4-2*, *cdfq* and *pif4-2 cdfq* grown under SDs for 7 days. Scale bar, 500 µm. Caption image shows representative transversal section of Col-0 hypocotyl. L, large non-dividing cell file; S, small dividing cell file. **b**,**c**,**d**, The mean cell number (**b**), cell length (**c**) and cell width (**d**) of the non-dividing cell files of hypocotyls. Box plots in panels **c** and **d** show the minimum, 25th percentile, median, 75th percentile and maximum of data points. Different letters in panels **b**, **c** and **d** represent significant differences among genotypes (*P* < 0.05), using ANOVA followed by Tukey’s pairwise multiple comparison, *P* = 0.325, 7.99 × 10^−7^ and 9.64 × 10^−5^ in **b**, **c** and **d**, respectively); *n* = 8 cell files examined over 4 hypocotyls, cell numbers are presented in Fig. 1b. Source data

To understand the histological differences underlying variation in hypocotyl length among these genotypes, the size and number of cells in the epidermis were measured. Non-dividing cell files were examined to assess the effect of the mutations on cell growth^27^ (Fig. 1a). Analysis of confocal microscopy images showed that the numbers of cells in the non-dividing files were highly similar among all genotypes (Fig. 1b), although the hypocotyls of *pif4*, *cdfq* and *pif4 cdfq* mutants were shorter than those of Col-0 (Extended Data Fig. 1a). In each genotype, the length of cells in the non-dividing files increased basipetally from the shoot apical meristem to the collet, particularly between cells 8 and 14 (Fig. 1a and Extended Data Fig. 1e), as described for dark-grown Col-0 seedlings^27^. In *pif4*, *cdfq* and *pif4 cdfq* mutants, the mean cell length in these files was shorter than in Col-0, particularly between cells 5 and 10 (Extended Data Fig. 1e), and there was no significant difference in cell length among the mutants (Fig. 1c). The cell width of the non-dividing files decreased basipetally (Extended Data Fig. 1f). The mean cell width in the non-dividing files in *pif4 cdfq* was slightly narrower than that of Col-0, *cdfq* and *pif4* plants (Fig. 1d). These histological analyses suggest that *PIF4* and the *CDF*s act in the same genetic pathway to promote elongation of hypocotyl cells in non-dividing files.

### CDF2 and PIF4 are co-expressed and physically interact

**Fig. 2:**
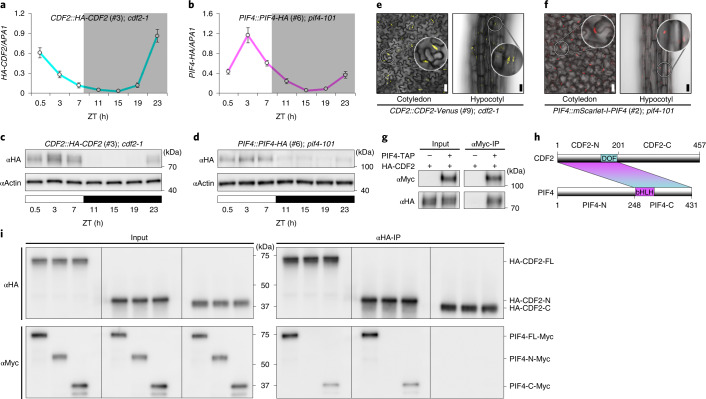
Spatio-temporal expression patterns of *CDF2* and *PIF4*, and physical interaction between the two proteins. **a**,**b**, RT–qPCR analysis of levels of *HA*-*CDF2* (**a**) and *PIF4*-*HA* (**b**) mRNA. Data are represented as means ± SEM of three independent amplifications. All values are normalized to *APA1* levels. **c**,**d**, Western blots comparing the accumulation of HA-CDF2 (**c**) and PIF4-HA (**d**) proteins. Time (h) is expressed as hours from dawn (ZT, zeitgeber). Actin served as the loading control. RNA and protein were extracted throughout a SD in 6-day-old seedlings. Western blots in **b** and **c** were performed twice with similar results. **e**,**f**, Confocal microscopy analysis of epidermal cells of 6-day-old *CDF2*::*CDF2*-*mVenus* (**e**) and *PIF4*::*mScarlet*-*I-PIF4* (**f**) transgenic plants grown under SD. Scale bars, 30 μm. **g**, HA-CDF2 protein co-immunoprecipitates with PIF4-TAP (9×Myc-6×His-3×Flag) from 6-day-old SD-grown seedlings. Co-IP experiments in **b** and **c** were performed twice with similar results. **h**, Box diagram of various fragments of CDF2 and PIF4 used in **i**. **i**, PIF4-Myc (C-terminal) interacts with HA-CDF2 (N-terminal) in vitro in the light. PIF4-Myc, HA-CDF2 and their truncation proteins were synthesized in a cell-free system. In vitro pull-down assays in **i** were performed three times with similar results. Source data

**Extended Data Fig. 2:**
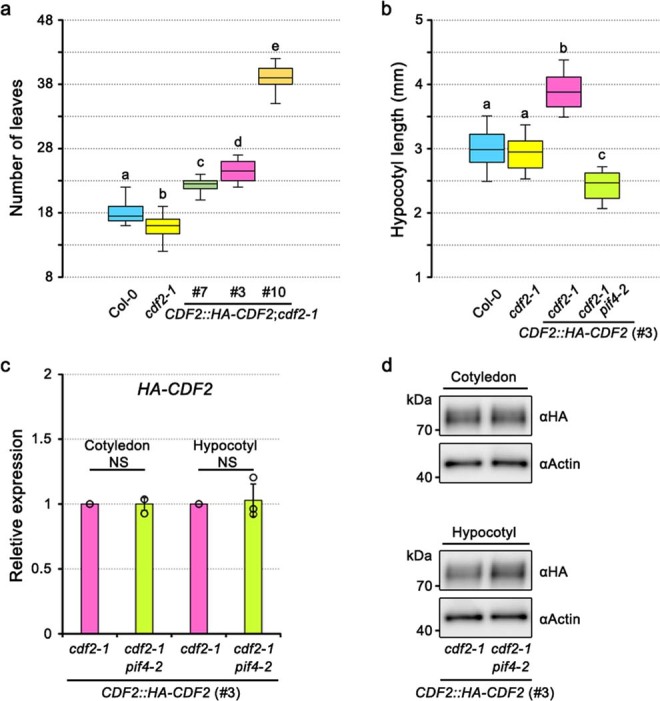
Flowering time and hypocotyl length of transgenic plants carrying *CDF2::HA-CDF2*. Flowering time and hypocotyl length of transgenic plants carrying *CDF2::HA-CDF2*. **a**, Flowering time of WT, *cdf2-1* and *CDF2::HA-CDF2* transgenic lines in long-day conditions. The total leaf number was counted for plants of WT, *cdf2-1*, and T_3_ homozygous populations of three independent transgenic lines. Data are presented as means ± SEM; *n* = 14 for WT, *cdf2-1*, #7 and #3 and *n* = 13 for #10 in **a**. Box plots in panel **a** show the minimum, 25^th^ percentile, median, 75^th^ percentile and maximum of data points. *n* = (14, 14, 14, 14, 14). Letters in panels **a** show significant differences between genotypes (*P* < 0.05, using one-way ANOVA followed by Tukey test, two sided), *P* = 7.254*10^−45^. **b**, Hypocotyl length of 9-day-old seedlings of WT, *cdf2-1*, *CDF2::HA-CDF2* (#3), *cdf2-1* and *CDF2::HA-CDF2* (#3) *cdf2-1 pif4-2* in short-day conditions. Box plots in panel **b** show the minimum, 25^th^ percentile, median, 75^th^ percentile and maximum of data points. *n* = (30, 29, 29, 30). Letters in panels **b** show significant differences between genotypes (*P* < 0.05, using one-way ANOVA followed by Tukey test, two sided), *P* = 1.298*10^−42^. **c**, RT-qPCR analysis of *HA*-*CDF2* mRNA levels in cotyledons and hypocotyls of *cdf2*-*1* or *cdf2*-*1 pif4*-*2* mutants. All values are normalized to *APA1* levels. Data are presented as mean values ± SEM of three biological replicates. Statistical significance was determined by pairwise one-sided *t*-test (*P* = 0.4964 and 0.3869 in cotyledon and hypocotyl, respectively). Significant *P* < 0.05. NS, not significant. **d**, Western blotting analysis of the abundance of HA-CDF2 protein in cotyledons and hypocotyls in *cdf2*-*1* or *cdf2*-*1 pif4*-*2* mutants. Actin was used as the loading control. Western blots represent one of three independent biological replicates. Tissues were harvested at ZT-0.5 to ZT-1 from 7-day-old SD-grown seedlings in **c** and **d**. Source data

The temporal and spatial expression patterns of PIF4 and CDF2 were then compared. In a functional transgenic *CDF2::HA*-*CDF2 cdf2*-*1* line (Extended Data Fig. 2a–c) grown under SDs, *HA-CDF2* messenger RNA (mRNA) and protein exhibited similar diurnal cycles to those described for endogenous *CDF2* (ref. ^11^), reaching maximum levels early in the light period (Fig. 2a,c). Under the same conditions, *PIF4*-*HA* mRNA and protein in transgenic *PIF4::PIF4-HA pif4-101* plants^28^ also showed diurnal rhythms (Fig. 2b,d). Notably, the diurnal patterns of PIF4-HA and HA-CDF2 overlapped early in the light period, and were subsequently co-expressed for several hours (Fig. 2c,d).

To visualize the spatial accumulation of CDF2 and PIF4, transgenic lines expressing CDF2-mVenus and mScarlet-I-PIF4 fluorescent protein fusions from their native gene promoters were generated in *cdf2-1* and *pif4-2* mutants, respectively. Signals of both CDF2-mVenus and mScarlet-I-PIF4 were detected in the nuclei of epidermal cells of cotyledons and hypocotyls (Fig. 2e,f), consistent with the induction of hypocotyl growth by the epidermal-specific expression of *PIF4* (ref. ^17^).

Whether PIF4 could physically interact with CDF2 in vivo was then tested. Plants that co-expressed *35S::PIF4-TAP* (*9Myc-6His-3Flag*)^22^ and *CDF2::HA-CDF2* were generated, and HA-CDF2 was co-immunoprecipitated with PIF4-TAP at ZT-0.5 in nuclear extracts from SD-grown seedlings (Fig. 2g). To understand the interaction domains between the two TFs, full-length CDF2 and PIF4 proteins as well as truncated versions were synthesized in a cell-free system attached to epitope tags (Fig. 2h). In vitro, PIF4-Myc and PIF4-C-Myc were co-immunoprecipitated with HA-CDF2-FL (full length) and HA-CDF2-N using an anti-HA antibody (αHA-IP). However, no immunoprecipitation was detected using HA-CDF2-C or PIF4-N-Myc. These results indicate that direct physical interaction occurred through the PIF4-C and CDF2-N-terminal regions, which contained the PIF4^bHLH^ and CDF2^Dof^ DNA binding domains, respectively (Fig. 2i). Collectively, these experiments demonstrate that CDF2 and PIF4 are spatially and temporally co-expressed, and that they interact in vivo and in vitro.

### PIF4 and CDF2 bind to and co-regulate common target genes

**Fig. 3:**
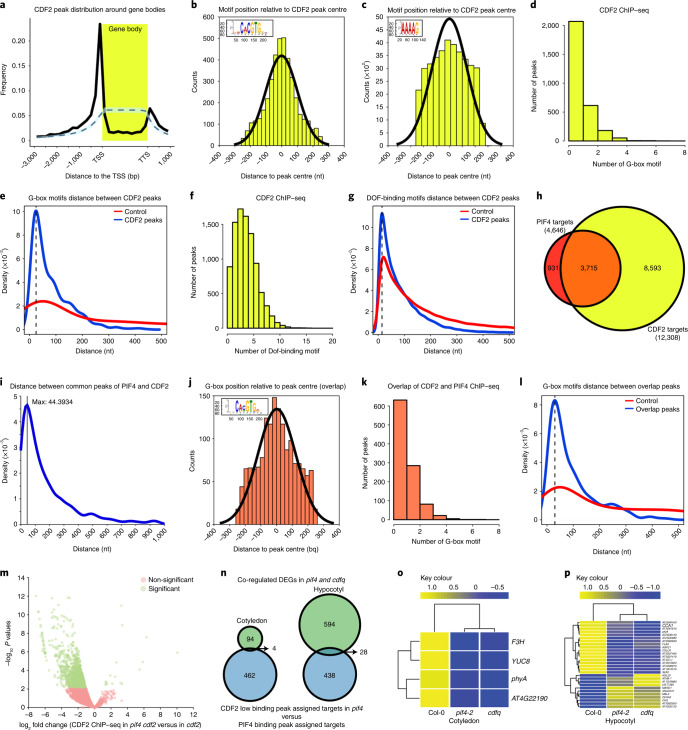
PIF4 and CDF2 bind to overlapping genomic targets and cooperatively regulate their expression. **a**, Positional distribution of CDF2 ChIP–seq peaks relative to the gene body. The observed distribution (black), the 95% confidence interval (shaded blue) and mean (dashed line) of 1,000 random peak sets are shown. **b**, Position distribution of G-box motifs relative to the CDF2 ChIP–seq peak centres. **c**, Position distribution of DOF-binding motifs relative to the centre of CDF2 ChIP–seq peaks. **d**, Frequency distribution of the number of G-box motifs observed in CDF2 ChIP–seq peaks. **e**, Density plots of the distance between consecutive G-box motifs in the observed CDF2 ChIP–seq peaks (blue) and the promoters of all non-CDF2 targets (red). **f**, Frequency distribution of the number of DOF motifs observed in CDF2 ChIP–seq peaks. **g**, Density of the distances between consecutive DOF motifs in observed CDF2 ChIP–seq peaks (blue) and the promoters of non-CDF2 targets (red). **h**, Overlap between target genes of CDF2 and PIF4. **i**, Density plot showing the distribution of distances between PIF4 and CDF2 ChIP–seq peaks. **j**, Positional distribution of G-box motifs relative to centres of PIF4/CDF2 common ChIP–seq peaks. **k**, Frequency of G-box motifs observed in ChIP–seq peaks shared between PIF4 and CDF2. **l**, Density distribution of the distances between consecutive G-box motifs in ChIP–seq peaks common to the PIF4 and CDF2 peak sets (blue). The distribution is compared with that obtained by examining the promoters of all non-targets (red). **m**, Volcano plot describing the differential binding analysis of DiffBind package, by plotting the log_2_ fold change in binding strength against the −log_10_ *P* value of the differential binding test. The confidence threshold: false discovery rate ≤ 0.05. **n**, Venn diagram consistency in the direction of gene expression change in *cdfQ* and *pif4* mutants relative to that in Col-0. **o**,**p**, Heatmap showing the *z*-score normalized expression values of selected genes in the cotyledon (**o**) and hypocotyl (**p**) of Col-0, *cdfQ* and *pif4-2* mutants.

**Extended Data Fig. 3:**
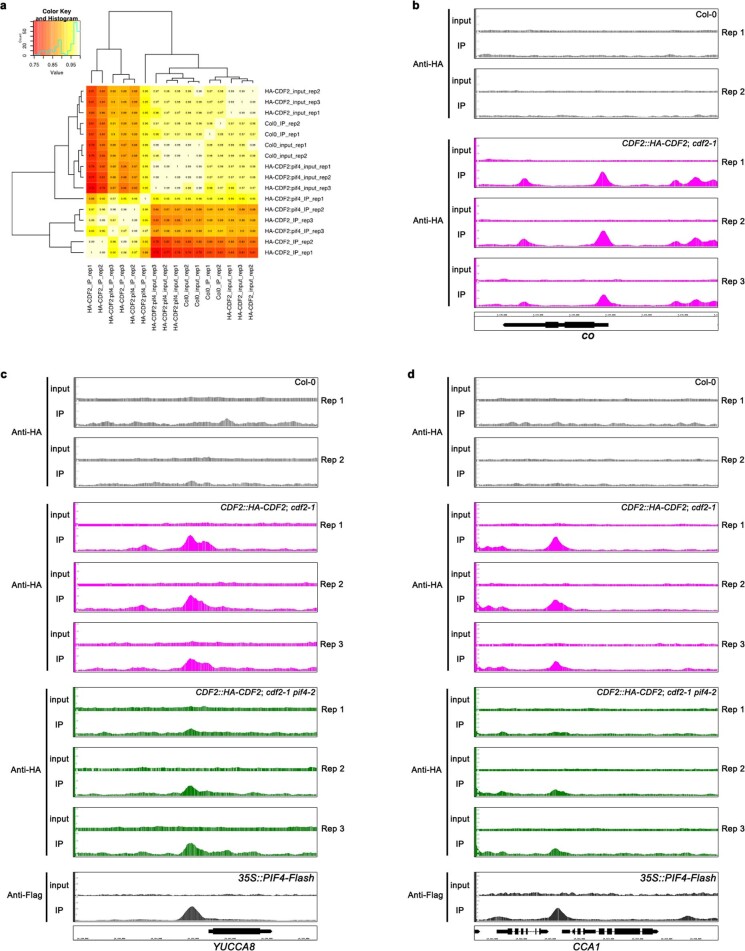
High reproducibility among the three biological replicates of ChIP-seq experiments with *CDF2::HA-CDF2* in *cdf2-1* and *cdf2-1 pif4-2* backgrounds. High reproducibility among the three biological replicates of ChIP-seq experiments with *CDF2::HA-CDF2* in *cdf2-1* and *cdf2-1 pif4-2* backgrounds. **a**, Pearson correlation between the read coverage along the genome for input and chromatin-immunoprecipitated (IP) samples. **b**, CDF2-binding profile for the *CONSTANS* (*CO*) gene. The panels display two biological replicates for the ChIP-seq control in Col-0 and three biological replicates for ChIP-seq of CDF2 to the *CO* locus, visualized with the Integrated Genome Browser (IGB). **c**, CDF2- and PIF4-binding profiles to *YUCCA8* and **d** to *CIRCADIAN CLOCK ASSOCIATED1* (*CCA1*). Each set of panels displays two biological replicates for the ChIP-seq control in Col-0, three biological replicates for ChIP-seq of CDF2 in *cdf2-1* and *cdf2-1 pif4-2* backgrounds, and a single biological replicate for ChIP-seq of PIF4^21^, visualized with the IGB.

**Extended Data Fig. 4:**
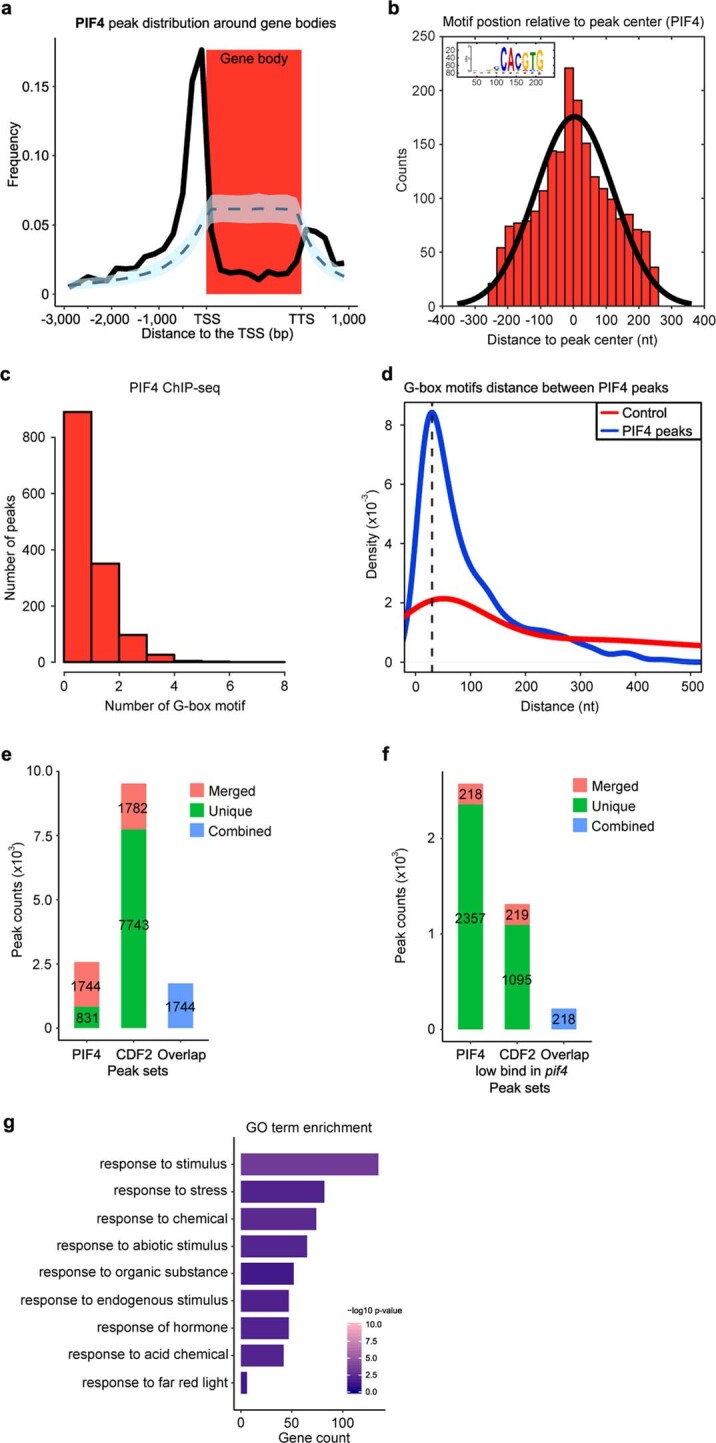
Re-analysis of PIF4 ChIP seq data. Re-analysis of PIF4 ChIP seq data. **a**, Positional distribution of PIF4-binding peaks on Arabidopsis genes. The observed distribution (black), the 95% confidence interval (shaded blue) and mean (dashed line) of 1,000 random peak sets are shown. **b**, Histogram showing the positional distribution of G-boxes relative to the peak centre (E-value = 3.5e-39). **c**, Histogram showing the number of PIF4 peaks with different numbers of G-box motifs. **d**, The distance between consecutive G-box motifs in a single PIF4 peak. The density of the distance between consecutive G-boxes in the promoters of all non-PIF targets was used as a control. **e**, Overlapping gene set between PIF4 and CDF2 peaks. Note that in a limited number of cases, multiple peaks in a single sample (merged peaks) overlap with a single peak in the other sample. **f**, Overlapping gene set between PIF4 peaks and CDF2 peaks with significantly reduced binding affinity in the *pif4* mutant. Similar to in **e**, multiple peaks in a single sample can overlap with a single peak in the other sample. **g**, GO-term enrichment based on the gene set between PIF4 peaks and CDF2 peaks with significantly reduced binding affinity in the *pif4* mutant. Enrichment tests were performed with topGO for each GO term separately using the Fisher Exact test. Raw *p*-values were adjusted using the Benjamini-Hochberg procedure. GO terms with adjusted *p*-values < 0.05 were considered significantly enriched.

PIF4 directly interacts with other TFs through their DNA-binding domains to recognize promoters of common target genes^4,29^. We performed chromatin immunoprecipitation sequencing (ChIP–seq) to identify the in vivo binding sites of HA-CDF2 and to compare these with previously identified PIF4 binding sites^21^. A total of 9,027 CDF2 binding peaks were identified and associated with 12,308 neighbouring genes (Supplementary information, Extended Data Fig. 3a and Supplementary Table 1). The majority (81.6%) of the peaks were within 3 kb of sequence 5′ to the transcription start site of a gene (Fig. 3a), consistent with the action of CDF2 as a transcriptional regulator. The canonical DOF-binding motif AAAAG was overrepresented (*E* value 1.7 × 10^−18^) in the centre of the ChIP–seq peaks (Fig. 3b,c), but the G-box (CACGTG), which is recognized by PIF4^19,21^, and closely related sequences were identified as the most enriched motifs (*E* value 8.5 × 10^−258^). About 20% of CDF2-binding peaks contained one G-box and approximately 9% contained more than one (Fig. 3d), with a peak in spacing distance of 25 bp (Fig. 3e). Similarly, 87% of CDF2-binding peaks contained two or more DOF-binding motifs, with a maximum of three per peak (Fig. 3f), and a most frequent spacing distance of 15 bp (Fig. 3g). To test whether PIF4 recognizes the G-boxes at CDF2 targets, we reanalyzed ChIP–seq data of PIF4^21^ (Supplementary Table 2). Similar to the findings of previous studies^4,21^, the highest frequency (88.19%) of PIF4 occupancy was located within 3 kb of sequence 5′ to the transcription start sites of genes (Extended Data Fig. 4a), and G-boxes were remarkably enriched (*E* value 3.5 × 10^−39^) in the centre of PIF4-occupancy regions (Extended Data Fig. 4b). More than 480 (19%) PIF4 peaks contained at least two closely spaced G-boxes (Extended Data Fig. 4c), and these motifs showed a most frequent spacing distance of 30 bp, similar to the arrangement of G-boxes found in CDF2 targets (Extended Data Fig. 4d and Fig. 3e).

The target genes and occupancy regions of these two TFs were then compared. The overlap among CDF2 and PIF4 target genes was highly significant (Fig. 3h and Supplementary Table 3) (*P* value < 2.2 × 10^−16^). In total, 1,744 common peaks of CDF2 and PIF4 were identified (Extended Data Fig. 4e), and were closely spaced (Fig. 3i). Within these common peaks, G-box motifs were significantly enriched (*E* value 6.1 × 10^−180^) in their centre (*E* value 2.8 × 10^−20^) (Fig. 3j), at a similar number and spacing to what was observed in all PIF4 peaks (Fig. 3k,l). Therefore, CDF2- and PIF4-binding sites are closely spaced in a common set of target genes.

To test whether genome occupancy by CDF2 requires PIF4, HA-CDF2 ChIP–seq was performed in the *pif4-2* mutant (Extended Data Fig. 3). A strong bias towards weaker binding of HA-CDF2 was detected in *pif4-2 cdf2-1* mutants compared with that in *cdf2-1* (Fig. 3m), which was not due to lower levels of mRNA or protein expression of the *HA*-*CDF2* transgene in *pif4-2 cdf2-1* (Extended Data Fig. 2d). We identified 1,314 peaks, of which 1,274 were assigned to 2,404 neighbouring genes (Supplementary Table 4) that showed differential binding of HA-CDF2 in *pif4-2 cdf2-1* compared with that in *cdf2-1* as determined using the DiffBind package (Methods). Approximately 16.6% (218 out of 1,314) of those peaks (Extended Data Fig. 4f), which were assigned to 466 genes (Supplementary Table 5), were also identified as being bound in the PIF4 ChIP–seq. Gene Ontology analysis of the differentially bound genes identified enrichment in several biological processes, including response to abiotic stimulus, response to far-red light, and response to hormones (Extended Data Fig. 4g), which were previously identified as highly represented in PIF4-mediated responses^4,17,21^.

**Extended Data Fig. 5:**
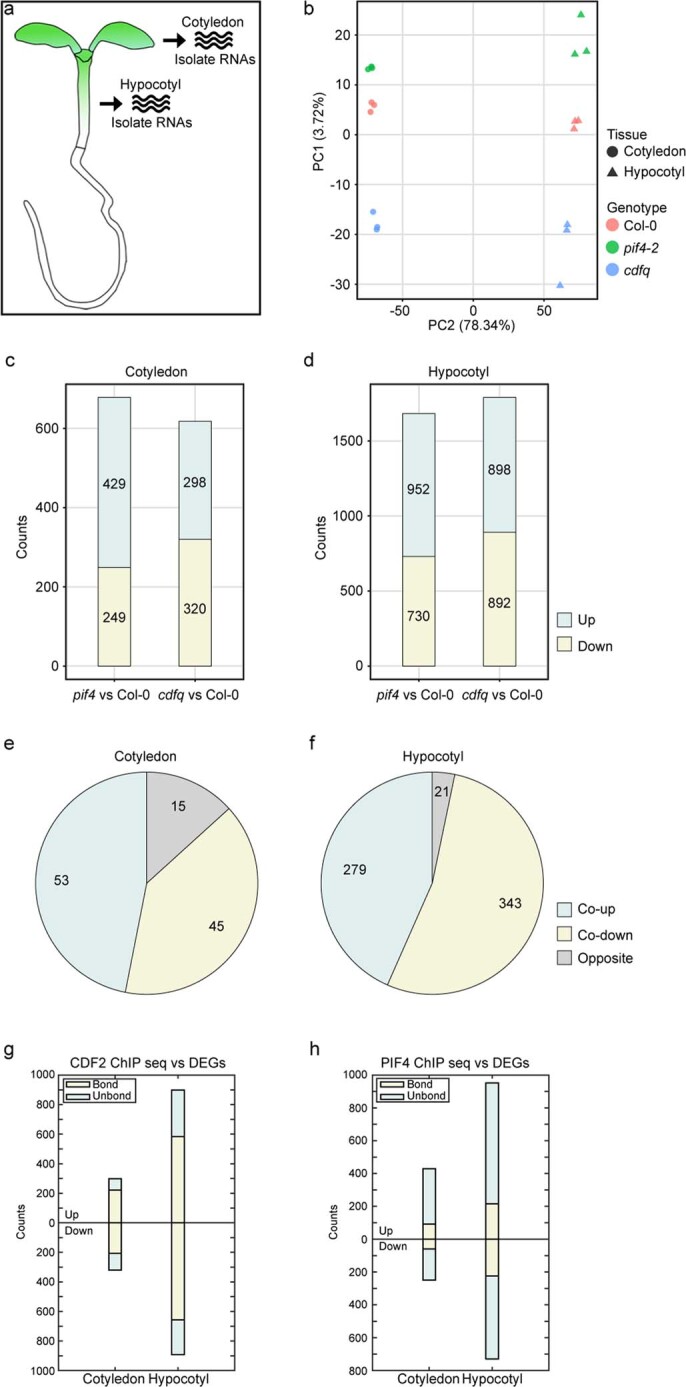
Transcriptome gene-expression profiling in experiments performed with different genetic backgrounds and tissues. Transcriptome gene-expression profiling in experiments performed with different genetic backgrounds and tissues. **a**, Twenty cotyledons and fifty hypocotyls were sampled for RNA-seq. **b**, The two major principal components (PC) from a PC analysis of the log_2_(FPKM + 1)-transformed gene expression levels in the different samples. **c** and **d**, The number of significantly up- and downregulated genes in the cotyledons (**c**) and hypocotyls (**d**) of *pif4* and *cdfq* mutants relative to the wild type. **e** and **f**, The number of genes showing the same and opposite directions of regulation in the cotyledon (**e**) and hypocotyls (**f**) of *pif4* and *cdfq* mutants relative to the wild type. A hypergeometric test showed that the probability of the overlap between the differentially expressed genes (DEGs) in *pif4* and *cdfq* for cotyledons and hypocotyls was 4.882719e-74 and 0, respectively, indicating that the difference in the expression level of genes in these transcriptomes is higher than expected by chance. **g**, Overlapping gene set between CDF2 targets and DEGs in the *cdfq* mutant relative to the wild type. **h**, Overlapping gene set between PIF4 targets and differentially expressed genes in the *pif4* mutant relative to the wild type.

To address how binding of PIF4–CDF2 regulates gene expression, we compared the transcriptomes of cotyledons and hypocotyls in Col-0, and in *pif4* and *cdfq* mutants grown under SDs by RNA-sequencing (RNA-seq) (Extended Data Fig. 5). Differentially expressed genes (DEGs) in *pif4-2* (Supplementary Table 6) or *cdfq* (Supplementary Table 7) mutants compared with Col-0 were identified using a threshold of 1.5-fold change with an adjusted *P* value < 0.05. Overall, more DEGs were identified in hypocotyls than in cotyledons (Extended Data Fig. 5d). Among the DEGs, 113 (*P* = 4.882719 × 10^−74^) in cotyledons and 643 (*P* = 0) in hypocotyls were shared between *pif4-2* and *cdfq* mutants (Extended Data Fig. 5e,f). In both tissues and genotypes, co-regulated genes were upregulated and downregulated at similar frequencies and only 13% of DEGs in cotyledons and 3% in hypocotyls showed opposite expression patterns in the two genotypes (Extended Data Fig. 5e,f and Supplementary Tables 8 and 9).

The target genes of CDF2 or PIF4 identified by ChIP–seq were compared with the DEGs in cotyledons and hypocotyls of each mutant (Extended Data Fig. 5g,h). Common DEGs that were bound by both PIF4 and CDF2 and that were bound less strongly by CDF2 in the *pif4* mutant background were then extracted. In this way, a stringent list was identified, containing 32 genes (4 in cotyledons and 28 in hypocotyls) that were considered with high confidence to be cooperatively regulated by PIF4 and CDF2 (Fig. 3n and Supplementary Table 10). Notably, they included *YUCCA8* (*YUC8*) (Fig. 3o and Extended Data Fig. 3c), which encodes an enzyme involved in rapid auxin biosynthesis in response to light signals and whose expression has been linked to the function of PIF TFs^19,30^, and in hypocotyls of *CIRCADIAN CLOCK ASSOCIATED 1* (*CCA1*) (Fig. 3p and Extended Data Fig. 3d), which encodes a MYB-related TF that acts in the morning to mediate circadian clock-regulated hypocotyl elongation^31^. Both of these genes were downregulated in *pif4* and *cdfq* mutants (Fig. 3o,p). The PIF4–CDF2 module therefore regulates different target genes in hypocotyls and cotyledons, and PIF4 enhances the strength of CDF2 association with DNA in vivo.

### Open chromatin at common targets of PIF4 and CDF2

**Fig. 4:**
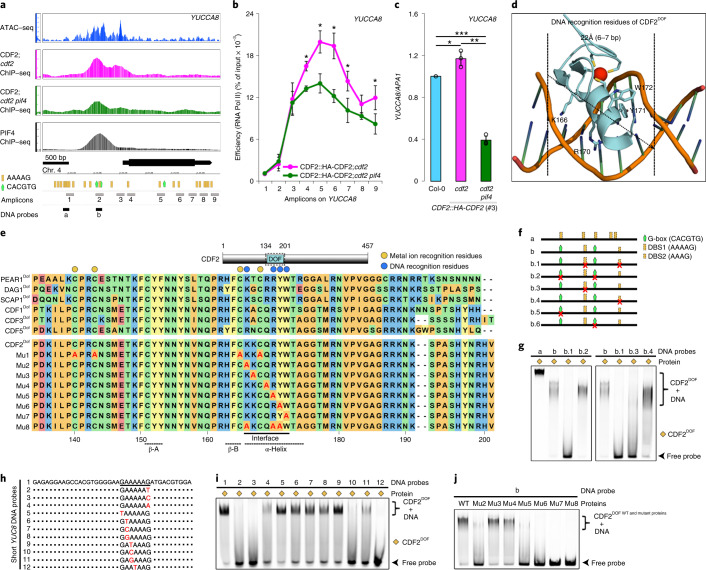
Mapping and functional cooperation of PIF4 and CDF2 binding in the *YUCCA8* promoter in vivo and overall structure modelling of the CDF2^DOF^–DNA complex. **a**, Windows for Assay for Transposase-Accessible Chromatin using sequencing (ATAC–seq) analysis^31^ and CDF2 and PIF4^21^ binding profile to the *YUCCA8* gene. The DOF-binding sites and G-boxes are shown through the whole gene body. The locations of amplicons for ChIP–qPCR analysis in **b** and probes for EMSA experiments in **f** and **g** are also shown. **b**, ChIP–qPCR analysis of RNAPII enrichment in the transcribed regions of *YUCCA8* in transgenic plants carrying *CDF2::HA-CDF2* in *cdf2* and *cdf2 pif4* backgrounds. The transcribed region of *YUCCA8* is marked with the black box that matches with positions of the analyzed amplicons in **a**. Data are represented as means ± SEM of three independent amplifications. Statistical significance was determined by pairwise one-sided *t*-test (for amplicons 1-8 in CDF2::HA-CDF2; *cdf2* versus CDF2::HA-CDF2; *cdf2 pif4*, *P* = 0.396, 0.112, 0.306, 0.047, 0.017, 0.024, 0.156 and 0.03). Asterisks mark significant differences, ^∗^*P* < 0.05. **c**, RT–qPCR analysis of *YUCCA8* mRNA levels in Col-0 WT (wild type) and transgenic plants carrying *CDF2::HA-CDF2* in *cdf2* and *cdf2 pif4* backgrounds. Data are represented as means ± SEM of three independent amplifications. All values are normalized to *APA1* levels. Statistical significance was determined by pairwise one-sided *t*-test (Col-0 versus CDF2::HA-CDF2; *cdf2*, *P* = 0.0328085, Col-0 versus CDF2::HA-CDF2; *cdf2 pif4*, *P* = 0.0010115 and CDF2::HA-CDF2; *cdf2* versus CDF2::HA-CDF2; *cdf2 pif4, P* = 0.0004875). Asterisks mark significant differences, ^∗^*P* < 0.05, ^∗∗^*P* < 0.01, ^∗∗∗^*P* < 0.001. **d**, Modelled structure of the CDF2^DOF^–DNA complex. **e**, DOF domain architecture of CDF2 and alignment of the DOF domain among CDF1, 2, 3, 5 and PEAR1, DAG1 and SCAP1 proteins in Arabidopsis. **f**, Overview of the long WT and mutant DNA probes (95 bp) for EMSA experiments for analysis of CDF2^DOF^ DNA-binding specificity and affinity. **g**, Interactions between CDF2^DOF^ protein and DNA probes analyzed by EMSA. **h**,**i**, Overview of short WT (**h**) and mutant DNA probes (38 bp) (**i**) within the long *YUCCA8* DNA probe, and interactions between CDF2^DOF^ and DNA probes analyzed by EMSA. **j**, Mutations in the predicted residues of the CDF2^DOF^ domain for DNA binding. Alignment among some Arabidopsis DOF TFs is shown in **e**. Interactions between DNA and CDF2^DOF^ WT and mutant proteins in the predicted residues of CDF2^DOF^ domain analyzed by EMSA. EMSA assays in **g**, **i** and **j** were performed three times with similar results. Source data

To understand in more detail how PIF4 and CDF2 coordinate transcriptional regulation, we focused on *YUC8*. Assay for Transposase-Accessible Chromatin using sequencing analysis showed that the binding peaks of PIF4 and CDF2 on *YUC8* (ref. ^32^) were located in an open chromatin region (Fig. 4a). By contrast, the G-box (CACGTG) in the coding region and other DOF-binding sites (AAAAG) located throughout the whole gene body were within closed chromatin regions and were not detected in the ChIP–seq of PIF4 and CDF2, respectively (Fig. 4a). The binding affinity of CDF2 in the *YUC8* promoter region was much reduced when PIF4 was absent (Fig. 4a and Supplementary Table 4), supporting the notion that PIF4 recruits CDF2 to their common targets. To initiate gene transcription, RNA polymerase II (Pol II) assembles with general initiation factors at the promoter regions of genes to form the pre-initiation complex. During pre-initiation complex assembly, the Mediator coactivator complex bridges upstream TFs and RNA Pol II^33^, and in tomato, PIF4 induces transcription via interaction with the Mediator subunit 25 (MED25)^34^. Similarly, the enrichment of RNA Pol II along the transcribed region of *YUC8* showed a significant decrease when PIF4 was absent (Fig. 4b), consistent with the lower level of *YUC8* mRNA in the *CDF2::HA-CDF2 pif4-2 cdf2-1* mutant (Figs. 3o and 4c).

### Molecular basis for CDF2^DOF^ binding to the *YUCCA8* promoter

**Extended Data Fig. 6:**
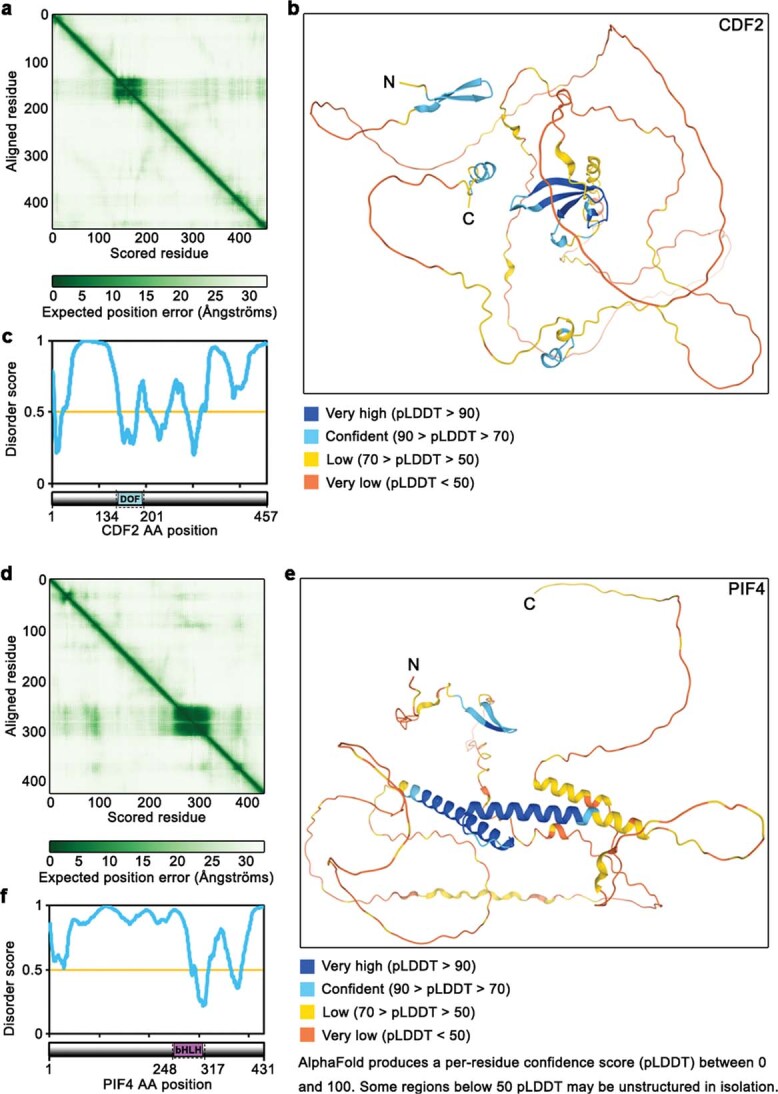
The predicted CDF2 and PIF4 protein structure. The predicted CDF2 and PIF4 protein structure. **a**, Predicted aligned error of CDF2. The colour at position (x, y) indicates AlphaFold’s expected position error at residue x, when the predicted and true structures are aligned on residue y. **b**, Overall view of predicted CDF2 protein with model confidence. **c**, Prediction of intrinsically disordered tendency of CDF2 (http://www.pondr.com/). **d**, Predicted aligned error of PIF4. The colour at position (x, y) indicates AlphaFold’s expected position error at residue x, when the predicted and true structures are aligned on residue y. **e**, Overall view of predicted PIF4 protein with model confidence. **f**, Prediction of the intrinsically disordered tendency of PIF4.

**Extended Data Fig. 7:**
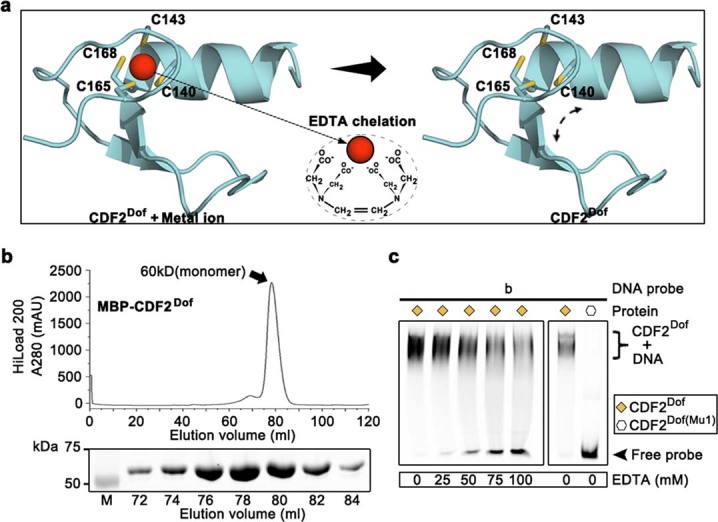
The putative interaction between a metal ion and the four cysteine residues stabilizes the structure of the CDF2^DOF^ domain in an appropriate conformation for DNA binding. The putative interaction between a metal ion and the four cysteine residues stabilizes the structure of the CDF2^DOF^ domain in an appropriate conformation for DNA binding. **a**, Modelled structure of the CDF2^DOF^ domain with the four cysteine residues (C140, C143, C165 and C168) highlighted. Removal of the metal ion by adding a divalent metal chelator destabilizes the structure. **b**, Size-exclusion chromatography analysis of CDF2^DOF^ WT protein. The CDF2^DOF^ domain is fused with an MBP tag at the N terminus. The x-axis and y-axis indicate the elution volume and the protein absorption at 280 nm, respectively. **c**, Gel-shift analysis of the interactions between CDF2^DOF^ WT, Mu1 proteins and DNA in various concentrations of EDTA. Size-exclusion chromatography analysis in **b** and EMSA assays in **c** were performed twice with similar results. Source data

CDF2 is predicted to be highly disordered and apart from the DOF DNA-binding domain, which is highly conserved in all members of the family (Fig. 4e)^6^, no other structured domain(s) were predicted by AlphaFold^35^ and I-TASSER Suite^36^ (Extended Data Fig. 6a–c). Therefore, to understand in more detail how it binds to DNA, a structural model of the CDF2^DOF^ domain was made based on the crystal structure of a zinc-finger (Zif268)–DNA complex^37^ (Methods and Fig. 4d). Similar to what is generally found in classical zinc-finger (ZF) proteins, one α-helix, which is inferred to contribute to DNA binding, and two β-sheets were also predicted in the CDF2^DOF^ domain (Fig. 4d,e). Similar to the results of previous studies^8^, our modelling of the CDF2^DOF^ domain showed that four cysteine residues (C140, C143, C165 and C168) in the conserved CX_2_CX_21_CX_2_C motif are likely to bind a metal ion (probably Zn) (Fig. 4d,e and Extended Data Fig. 7a). To stabilize the CDF2 DOF DNA-binding domain (CDF2^DOF^), an N-terminal maltose binding protein (MBP) was fused with it and the MBP-CDF2^DOF^ protein was purified from *Escherichia* *coli* (Methods). Gel-filtration results showed that MBP-CDF2^DOF^ protein was purified as a monomer (Extended Data Fig. 7b). To address the specific binding of CDF2^DOF^ to DNA, electrophoretic mobility shift assays (EMSA) were performed using DNA probes from the *YUC8* promoter (Fig. 4a,f). Fragment ‘a’, which contains five AAAAG motifs, was not bound by CDF2 in vivo (Fig. 4a), but was strongly bound by CDF2^DOF^ in vitro (Fig. 4f,g), supporting the notion that an in vivo open chromatin status is critical for accessibility of CDF2 to DNA. Fragment ‘b’, which is within the CDF2- and PIF4-binding peak regions on *YUC8* (Fig. 4a) and contains two G-boxes in addition to one AAAAG and one AAAG motif, was also bound by CDF2^DOF^ (Fig. 4f,g). Mutation of the G-boxes did not affect binding of CDF2^DOF^ (Fig. 4f,g), indicating that CDF2^DOF^ does not interact with the G-box directly in vitro. By contrast, mutation of both the AAAAG and AAAG motifs abolished CDF2^DOF^ binding, whereas mutation of the single motifs demonstrated that AAAAG was bound by CDF2^DOF^ much more strongly than AAAG (Fig. 4f,g). The EMSA assay was then used to further identify the base pairs that are bound by CDF2^DOF^. The results indicated that the 5-bp core of the DOF-binding motif [T/A]AAAG led to the maximum binding affinity, explaining why the AAAAG motif in *YUC8* is recognized more efficiently than the AAAG motif, and that the position of the 3′ G nucleotide is critical for the strength of binding of CDF2^DOF^ to DNA in vitro (Fig. 4h,i).

We then tested the structural model by mutating the CDF2^DOF^ protein sequence. Binding of the metal ion to the four cysteines was predicted to strongly stabilize the CDF2^DOF^ domain in an appropriate conformation for DNA interaction. Consistent with this, adding divalent metal chelator ethylenediaminetetraacetic acid (EDTA) or mutating the four cysteines (C140, C143, C165 and C168) to alanine (CDF2^DOF (Mu1)^) diminished or abolished interaction between CDF2^DOF^ and DNA (Fig. 4e and Extended Data Fig. 7c). The structural model showed that the α-helix of CDF2^DOF^ could fit into the DNA major groove (22 Å), and contribute to DNA binding. Mutations in Y171 (Mu6: Y171A) and W172 (Mu7: W172A) within the α-helix abolished DNA binding (Fig. 4d,e,j), as observed previously for conserved residues in DOF AOBP (ascorbate oxidase promoter-binding protein)^38^, and consistent with these residues contributing to DNA recognition. However, our modelled CDF2^DOF^–DNA complex suggested that additional residue(s) in the putative α-helix might interact with DNA (Fig. 4d). Mutation of K167 (Mu3: K167A) and Q169 (Mu4: Q169A) had no detectable effect on binding, but mutation of K166 (K166A: Mu2) and R170 (R177A: Mu5) strongly reduced DNA binding (Fig. 4e,j). These two conserved residues, which were recently identified to be important for DNA binding, are very close to C165 and C168 and conformational changes induced by metal binding might influence their accessibility to DNA. Similar to Mu7, mutation of the three residues in the α-helix of the CDF2^DOF^ domain (Mu8: K166, R170 and Y171) abolished its DNA-binding function (Fig. 4d,e,j). Taken together, these data confirmed the known residues (Y171 and W172) and identified additional residues (K166 and R170) involved in DNA binding, extending the interface necessary for DNA binding in vitro and supporting the structural model, which indicated that these residues of CDF2^DOF^ interact with the five-nucleotide AAAAG motif within the major groove.

### Molecular basis for PIF4^bHLH^ binding to the *YUCCA8* promoter

**Fig. 5:**
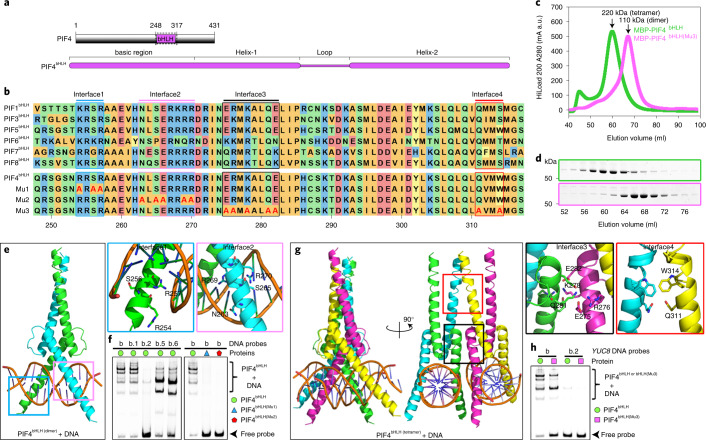
Overall structure modelling of the PIF4^bHLH^–DNA complex. **a**,**b**, Domain architecture of PIF4 (**a**) and alignment of bHLH domain among PIF1, 3, 4, 5, 6, 7 and 8 proteins (**b**) of Arabidopsis. The dotted box on PIF4 indicates the protein boundaries used for structure modelling and gel-shift assays. **c**, Size-exclusion chromatography analysis of PIF4^bHLH^ WT and mutant (Mu1) proteins. PIF4^bHLH^ domains were fused with an MBP tag at the N terminus. The *x* axis and *y* axis indicate the elution volume and protein absorption at 280 nm. The WT PIF4^bHLH^ and Mu1 proteins are coloured in green and pink, respectively. a.u., arbitrary units. **d**, Eluted protein samples from the same peak fractions of WT PIF4^bHLH^ and Mu3 proteins from **c**, were subjected to SDS–PAGE followed by Coomassie blue staining. Size-exclusion chromatography analysis in **d** was performed twice with similar results. **e**, Modelled structure of the DNA-bound PIF4^bHLH^ homodimer and zoom-in of the predicted PIF4^bHLH^ residues that interact with DNA are coloured in blue (Interface 1) and pink (Interface 2). **f**, Gel-shift analysis of the interactions between PIF4^bHLH^ WT, Mu1 and Mu2 proteins and DNA probes (as described in Fig. 4f). **g**, Modelled structure of the DNA-bound PIF4^bHLH^ homotetramer and a zoom-in of the predicted PIF4^bHLH^ residues that contribute to tetramer formation are coloured in black (Interface 3) and red (Interface 4). **h**, Gel-shift analysis of the interactions between PIF4^bHLH^ WT, Mu3 proteins and DNA probes (as in Fig. 4f). Alignment of the two predicted DNA-interacting interfaces and the two protein-interacting interfaces among the Arabidopsis PIF proteins and residue mutations of those interfaces on PIF4^bHLH^ are shown in **b**. EMSA assays in **f** and **h** were performed three times with similar results. Source data

**Extended Data Fig. 8:**
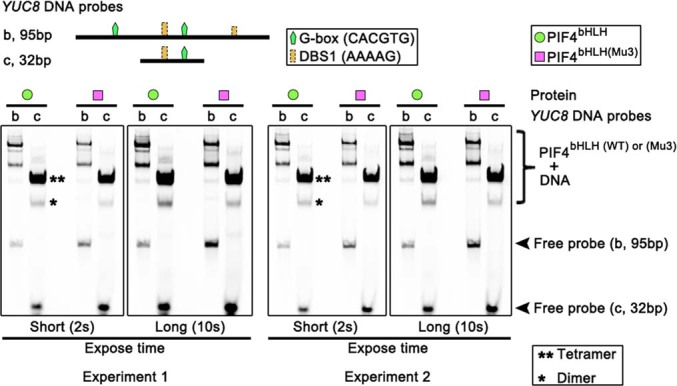
Gel-shift analysis of the interactions between PIF4^bHLH^ WT protein and different length of DNA probes. Gel-shift analysis of the interactions between PIF4^bHLH^ WT protein and different length of DNA probes (as described in Fig. 4f). Several different sizes of complexes of PIF4^bHLH^ bound to the longer DNA fragment, ‘b’ could be reduced using shorter DNA fragments (fragment ‘c’) containing only one G-box. EMSA assays were performed twice with similar results.

**Extended Data Fig. 9:**
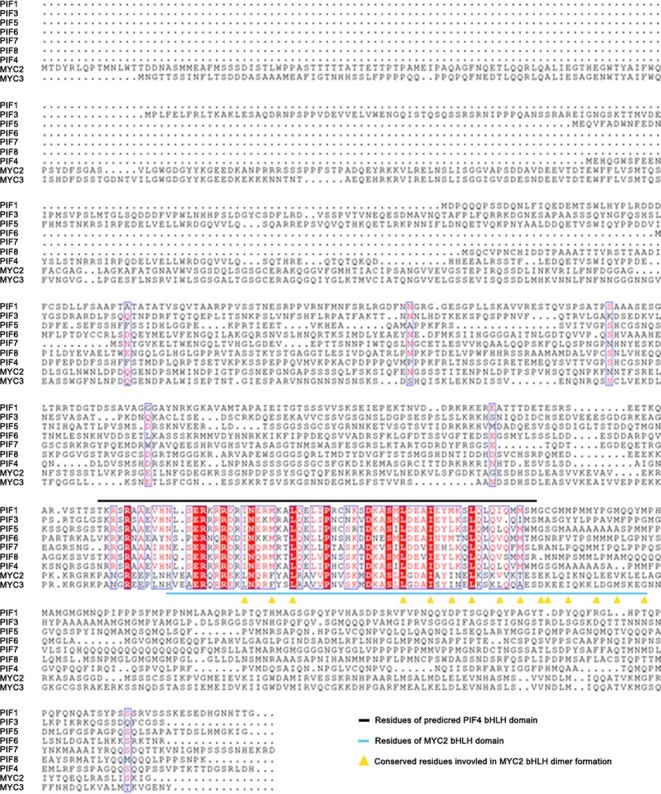
A multiple sequence alignment for PIFs and MYC2 and MYC3 proteins. A multiple sequence alignment for PIFs and MYC2 and MYC3 proteins (https://espript.ibcp.fr/ESPript/ESPript/). Black bar, residues of predicted PIF4^bHLH^ domain. Light blue bar, residues of MYC2 bHLH domain. Yellow triangle, residues involved in MYC2^bHLH^ dimer formation.

Similar to CDF2, structural modelling predicted that PIF4 protein is not well ordered (Extended Data Fig. 6d–f), except for the bHLH–DNA-binding domain, which showed a strong preference for binding the G-box (5′-CACGTG-3′)^4,18,19,21^ (Fig. 5a,b). To further understand the molecular basis of the interaction between the DNA-binding domain of PIF4 (PIF4^bHLH^) and DNA, we performed protein structure modelling based on the crystal structure of the MYC2 bHLH–DNA complex with G-box^39^ (Methods). This approach predicted that PIF4^bHLH^ binds DNA as a homodimer and two interfaces in the basic region of PIF4^bHLH^ bind DNA (Fig. 5b,e). To verify our structural modelling, PIF4^bHLH^ was purified via an N-terminal MBP fusion that conferred greater solubility on the protein. EMSA experiments were performed with fragment ‘b’ that was used previously for the CDF2^Dof^ experiments (Fig. 4f,i), and complexes of PIF4^bHLH^ bound to DNA of several different sizes were detected (Fig. 5f). This complexity was largely due to the presence of two G-boxes in the fragment because it could be reduced by using shorter DNA fragments containing only one G-box (Extended Data Fig. 8) or by mutation of single G-boxes (Fig. 5f). Mutation of both G-boxes in fragment ‘b’ demonstrated that PIF4^bHLH^ bound specifically to the G-boxes in the *YUC8* promoter (Fig. 5f). Moreover, mutations in Interface 1 (R254A, S256A and R257A; PIF4^bHLH (Mu1)^) or Interface 2 (N263A, S265A, S266A, R269A and R270AA; PIF4^bHLH (Mu2)^) prevented binding to DNA (Fig. 5b,e,f), consistent with the results of previous studies on PIF3 (ref. ^40^). The HLH domain induces homo- and heterodimerization between different PIFs^41^. Gel-filtration experiments demonstrated that MBP-PIF4^bHLH^ tended to form a homotetramers in solution (Fig. 5c,d). Previously, MYC2, a bHLH TF that functions in jasmonate signalling, was shown to form tetramers that enhanced DNA-binding strength, whereas MYC3 only formed dimers^39^. A multiple sequence alignment of the bHLH domains of PIFs, MYC2 and MYC3 was constructed to compare residues involved in dimerization. Most of the MYC2 bHLH residues involved in dimer formation are conserved in PIF bHLH domains, although a similar degree of conservation was observed in MYC3 and several residues varied at the C-terminus of the bHLH domains (Extended Data Fig. 9). No conserved residues associated with tetramerization could therefore be identified. To examine further the tetramerization of PIF4^bHLH^ in solution, we modelled PIF4 bHLH homotetramer based on MYC2–DNA complex structure^39^ (Fig. 5g), and a mutant protein (PIF4^bHLH (Mu3)^) was designed to impair interaction between the two dimers of PIF4^bHLH^ and thereby prevent tetramerization. Gel-filtration results showed that simultaneously mutating Interface 3 (E275A, R276A, K278A, Q281A and E282A) and Interface 4 (Q311A and W314A) abolished tetramerization of PIF4^bHLH^ such that the protein formed exclusively dimers (Fig. 5b–d,g), and this caused a reduction in DNA-binding affinity (Fig. 5h). Therefore, tetramerization between two PIF4^bHLH^ dimers enhances their DNA-binding affinity, probably because the tetramer can bind two adjacent G-boxes and bend DNA, as described previously for MYC2 (ref. ^39^).

### Binding of PIF4^bHLH^ to *YUCCA8* enhances CDF2^DOF^ binding

**Fig. 6:**
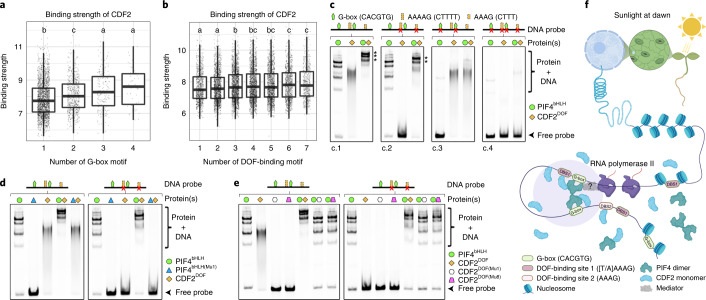
Binding of PIF4 on the G-boxes enhances CDF2-binding strength on DNA. **a**, Binding strengths for CDF2 peaks varied significantly with the numbers of G-box motifs (CACGTG, Kruskal–Wallis test, *P* = 4.588 × 10^−15^). **b**, Binding strengths for peaks with different numbers of DOF-binding motifs (AAAAG, Kruskal–Wallis test, *P* = 1.964 × 10^−7^). Box plots in panel **a** and **b** show the minimum, 25th percentile, median, 75th percentile and maximum of data points. Letters in panels **a** and **b** show significant differences among groups (adjusted *P* < 0.05) using pairwise Wilcox tests. Groups that share at least one identical letter are not significantly different. **c**, Gel-shift analysis of the interactions between single PIF4^bHLH^ or CDF2^DOF^ or combination of PIF4^bHLH^ and CDF2^DOF^ WT proteins with WT or mutant DNA probes. **d**, Gel-shift analysis of the interactions between single PIF4^bHLH^ or CDF2^DOF^ or combination of PIF4^bHLH^ WT or PIF4^bHLH (Mu1)^ mutant proteins and CDF2^DOF^ WT protein with WT or mutant DNA probes. **e**, Interactions between single PIF4^bHLH^ or CDF2^DOF^ or combinations of PIF4^bHLH^ WT and CDF2^DOF^ WT or CDF2^DOF (Mu1 and Mu8)^ mutant proteins with WT or mutant DNA probes analyzed by EMSA. EMSA assays in **c**, **d** and **e** were performed three times with similar results. **f**, Proposed model for the role of the PIF4–CDF2 module in regulating gene transcription in the light. Upon chromatin opening, the E-box and DOF-binding motifs are accessible. Binding of the G-boxes by PIF4, and the DOF-binding motifs near those G-boxes by CDF2 occurs. Interaction between PIF4 and CDF2 can occur when they are bound to DNA. Binding of PIF4 to the G-boxes strengthens CDF2 binding and allows it to bind to DNA independently of the DOF-binding sites. Therefore, PIF4 recruits CDF2 to the *YUCCA8* promoter. After binding of the PIF4–CDF2 module to chromatin, RNA polymerase II is recruited by PIF4 via the Mediator complex to induce gene transcription.

**Extended Data Fig. 10:**
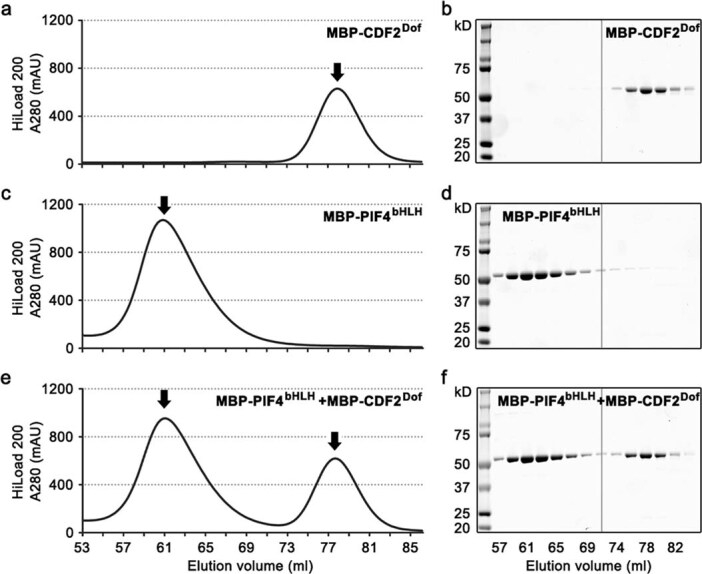
Size-exclusion chromatography analysis of CDF2^DOF^ and PIF4^bHLH^ WT proteins. Size-exclusion chromatography analysis of CDF2^DOF^ and PIF4^bHLH^ WT proteins. **a** and **b**, Size-exclusion chromatography and gel analysis of CDF2^DOF^ WT protein. **c** and **d**, Size-exclusion chromatography and gel analysis of PIF4^bHLH^ WT protein. **e** and **f**, Size-exclusion chromatography and gel analysis of CDF2^DOF^ and PIF4^bHLH^ WT proteins. The CDF2^DOF^ domain or PIF4^bHLH^ was fused with an MBP tag at the N terminus. The x-axes and y-axes for **a**, **c** and **e** indicate the elution volume and the protein absorption at 280 nm, respectively. Size-exclusion chromatography analysis in **b**, **d** and **f** were performed twice with similar results.

ZF proteins usually contain several tandemly arranged ZF motifs that strengthen interaction of the protein with DNA. CDF2 and other DOF proteins contain only a single ZF motif. However, the ChIP–seq analysis showed that CDF2 binding was highly correlated with the presence of G-boxes (Fig. 6a) but less so with AAAAG motifs (Fig. 6b), suggesting that PIF4 may enhance binding of CDF2 to adjacent AAAAG motifs, and thereby strengthen its specificity for particular genomic regions. On the other hand, PIF4 might also alter CDF2 specificity, because not all CDF2-binding regions that contained G-boxes also contained an adjacent AAAAG motif. Although the precise mechanism remains unknown, interaction between the two TFs might have a role in both scenarios. The involvement of protein interaction is consistent with the CDF2–PIF4 interaction observed in vivo and in vitro, which could occur through CDF2-N-terminal (1–201 amino acids (aa)) and PIF4-C-terminal (248–431 aa) regions (Fig. 2i). However, interaction between the CDF2^DOF^ (134–201 aa) and PIF4^bHLH^ (248–317 aa) domains used in the EMSA experiments was not detected by gel-filtration in vitro (Extended Data Fig. 10) indicating that the DNA-binding domains alone do not interact or do so very weakly. To test whether PIF4^bHLH^ enhanced binding of CDF2^DOF^ in vitro independently of strong interaction between them, EMSA was performed with the PIF4^bHLH^ and CDF2^DOF^ domains in combination. A supershift was detected with both proteins (Fig. 6c.1), indicating that they can bind to the same *YUCCA8* fragment. Unexpectedly, a supershift was still detected when the AAAAG and AAAG motifs were mutated, although to a lesser extent (Fig. 6c.2). By contrast, the supershift was strongly reduced when both CACGTG G-boxes were mutated (Fig. 6c.3). No shift or supershift was detected when both G-boxes and DOF-binding motifs were mutated (Fig. 6c.4). These results indicate that PIF4^bHLH^ binding to G-boxes is a determinant for the supershift, but that the DOF-binding motifs are not required. Consistently, the supershift was significantly reduced when mutant PIF4^bHLH (Mu1)^ protein that cannot bind DNA was combined with wild-type DNA probe and CDF2^DOF^ (Figs. 5 and 6d,left). Also, use of PIF4^bHLH (Mu1)^ abolished the supershift observed with PIF4^bHLH^ and CDF2^DOF^ on the mutated DOF-binding motif DNA (Fig. 6d, panel right). Furthermore, no supershift was detected when PIF4^bHLH^ was combined with CDF2^DOF (Mu1)^ or CDF2^DOF (Mu8)^, regardless of the presence of the DOF-binding motif (Fig. 4 and Extended Data Fig. 6). Therefore, the α-helix that is required for DNA binding by CDF2^DOF^ (Fig. 4) is required for the supershift with PIF4^bHLH^, even for DNA fragments that do not contain the AAAAG and AAAG motifs, and although these two truncated proteins do not detectably interact in vitro. These results suggest that binding of PIF4^bHLH^ to DNA induces other potential DNA interaction interface(s) of CDF2^DOF^ to access DNA, a process related to DNA allostery that was previously described^42^.

## Discussion

Hypocotyl cell elongation in the light requires cooperation between CDF2 and PIF4 to strongly activate transcription of *YUC8* in cotyledons, consistent with the previous observation that increased auxin biosynthesis in cotyledons through the action of YUCCA enzymes promotes hypocotyl growth^43^. We show that in vivo, CDF2binding strength and specificity are increased in the presence of PIF4 at a subset of common target genes, including *YUC8*, and this probably involves direct interaction between the proteins. Furthermore, in vitro, in the presence of PIF4^bHLH^, CDF2^DOF^ binds to a DNA fragment in which both DOF-binding sites are mutated, and as interaction of these two truncated proteins was undetectable by gel-filtration, this suggests that the binding of PIF4^bHLH^ may enhance the general affinity of CDF2^DOF^ for PIF4^bHLH^–DNA complex by DNA allostery^42^. TFs recognize their binding sites by directly interacting with specific bases, and by recognizing features of local DNA shape, such as DNA bending or unwinding^5^. Thus, we propose that PIF4 increases the strength and specificity of CDF2 DNA binding through protein–protein interactions that enhance sequence-specific DNA binding, and by altering local DNA shape. Whether CDFs influence PIF4 binding in vivo remains to be tested. We demonstrated that PIF4 forms tetramers and these may facilitate DNA looping, as demonstrated for MYC2 tetramers^39^, and thereby strengthen CDF2 binding at adjacent sites. The combinatorial interactions between PIF4 and CDF2 that we elucidated contribute to auxin biosynthesis and hypocotyl elongation in the light. Our results also enhance understanding of the transcriptional code that regulates plant gene expression in response to light and how this contributes to cell elongation.

## Methods

### Plant material and growth conditions

The *Arabidopsis thaliana* Columbia (Col-0) ecotype was used as the main experimental organism. Seeds of Col-0, *pif4*-*2* (SAIL_1288_E07), *cdf1i,2,3,5* (*cdfq*)^11^ and *pif4-2 cdfq* were surface-sterilized with 70% ethanol for 10 min, rinsed with 99% ethanol for 5 min, air-dried and stratified at 4 °C for 3 days. Plants were grown on soil under SD conditions (8 h light/16 h dark cycles) or were grown vertically on plates containing 1% agar supplemented with half-strength Murashige and Skoog medium (pH 5.7) at 22 °C with a light intensity of 160–180 μmol m^−2^ s^−1^ provided by LED bulbs (Philips F17T8/TL841 17 W). *cdf2*-*1* mutant plants were grown in the greenhouse under long-day conditions (16 h light/8 h dark cycles) and were transformed by the floral dip method using *Agrobacterium tumefaciens* strain GV3101.

### Hypocotyl length measurement

After stratification at 4 °C for 3 days, seeds were exposed to white light (at 160–180 μmol m^−2^ s^−1^) for 4 h to stimulate germination. Dark-grown plants were then kept in the dark for 4 days and SD plants were grown for 9 days before photos were taken for hypocotyl length measurements.

### Confocal imaging and cell segmentation

Hypocotyls of 7-day-old seedlings grown in SDs were dissected and fixed with 4% paraformaldehyde. The fixed samples were washed twice for 5 min in phosphate-buffered saline and cleared with ClearSee solution^44^ for 5 days in the dark at room temperature. The cell wall was stained with Renaissance 2200 (0.1% (v/v) in ClearSee)^45^ for 2 days. Confocal microscopy was performed using a TSC SP8 confocal microscope (Leica) as described previously^46^. The Renaissance excitation and image collection wavelengths were 405 nm and 410–503 nm, respectively. The interval between z-stack sections was 1 µm for maximum intensity projections and optical sections. The z-stacks of hypocotyl images were converted to .tiff files with Fiji. The surface of the hypocotyl was extracted using MorphoGraphX (MGX) software (https://morphographx.org/)^47^ and the Renaissance signal of the cell wall from the outer cell layer (L1) was projected and used to segment the images. Cells were automatically segmented and then corrected manually and the geometry of the surface was displayed as Gaussian curvatures.

### Generation of transgenic plants

To generate *pCDF2::3HA-CDF2*, the *CDF2* promoter and the full-length coding region were amplified from Col-0 genomic DNA and 3HA was amplified from plasmid pALLIGATOR2 using PrimeSTAR GXL DNA Polymerase (Takara Bio), then overlap PCR was performed using *CDF2* promoter, *3HA* and *CDF2* coding-region fragments. The *pCDF2::3HA-CDF2* fragments were cloned into the binary vector PER8-GFP by *Spe*I and *Xho*I digestion using an In-Fusion HD Cloning Kit (Takara Bio). The construct was delivered into *cdf2-1* via *Agrobacterium* GV3101 transformation using the floral dip method.

### Immunoblot assays

For SD time-course western blots, about 20 mg of tissue from 6-day-old seedlings was ground into fine powder in liquid nitrogen with a TissueLyser system (QIAGEN). Total proteins were extracted using denaturing buffer (100 mM Tris-HCl pH 7.5, 100 mM NaCl, 30 mM EDTA (ethylenedinitrilotetraacetic acid, Sigma-Aldrich) pH 8.0, 4% SDS (sodium dodecyl sulfate, Sigma-Aldrich), 20% glycerol, 20 mM β-mercaptoethanol (Sigma-Aldrich), 20 mM DTT (DL-dithiothreitol, Sigma-Aldrich), 2 mM PMSF (phenylmethylsulfonyl fluoride, Sigma-Aldrich), 1× Protease Inhibitor Cocktail (Sigma-Aldrich), 80 μM MG132 (Sigma-Aldrich), 1% Halt Phosphatase Inhibitor Cocktail (Thermo Fisher Scientific) and 0.01% bromophenol blue) in a 1:5 tissue:buffer (w/v) ratio by boiling for 10 min at 90 °C. Samples were centrifuged at 16,000*g* for 5 min at room temperature and the supernatants were electrophoresed on a sodium dodecyl sulfate–polyacrylamide gel electrophoresis (SDS–PAGE) mini-gel to separate the proteins.

For immunoblotting the separated proteins were transferred onto a polyvinylidene fluoride membrane by the Trans-Blot Turbo Transfer System (Bio-rad). Blots were probed with anti-HA (12013819001, Roche) or anti-Actin (sc-47778, Santa Cruz) antibodies conjugated to horseradish peroxidase (HRP). The blots were developed with a 1:1 mix of SuperSignal West Femto Maximum Sensitivity and SuperSignal West Dura Extended Duration Substrates and signals were detected on a ChemiDoc MP Imaging System (Bio-rad). Anti-HA (HRP) and anti-Actin (HRP) antibodies were used at 1:2,000 and 1:4,000-fold dilutions, respectively.

### In vitro pull-down assays

To express proteins in a cell-free system, 2HA-CDF2, PIF4-6Myc and the truncated DNA fragments were amplified by overlap PCR, then cloned into *Eco*RI-digested pTnT vector with the In-Fusion HD Cloning Kit (Takara Bio). For each construct (2HA-CDF2, PIF4-6Myc and truncated proteins (CDF2-N, 1–201 aa; CDF2-C, 202–457 aa; PIF4-N, 1–248 aa; PIF4-C, 249–431 aa)), 1.0 μg plasmid was expressed via the SP6 promoter in a cell-free system at 30 °C for 2 h in a thermocycler using the TnT Coupled Wheat Germ Extract System (Promega), according to the manufacturer’s instructions. A small amount of the reaction (2 μl) was used to verify expression of the target proteins by western blotting and the remaining extract (48 μl) was ‘snap-frozen’ in liquid nitrogen and stored at −80 °C.

For protein pull-downs, 60 μl extract (30 μl of 2HA-CDF2 or 30 μl of 2HA-CDF2 and 30 μl of PIF4-6Myc) was mixed with 540 μl IP buffer (22 mM Tris-HCl, pH 7.5; 84 mM NaCl; 1.1 mM EDTA; 0.11% Triton X-100 and 1× Plant Protease Inhibitor Cocktail (Sigma)) and rotated in the dark at 4 °C for 30 min. To pull down proteins, 30 μl of Dynabeads Protein G beads (Thermo Fisher Scientific) coated with 10 μl anti-Myc antibody (Cell Signaling Technology) was added to the diluted protein solution and was rotated for 30 min at 4 °C. The beads were washed five times with wash buffer (20 mM Tris-HCl, pH 7.5; 150 mM NaCl; 1 mM EDTA; 0.5% Triton X-100 and 0.1× Plant Protease Inhibitor Cocktail). Proteins were eluted from the beads with 2× SDS–PAGE sample buffer and then subjected to immunoblotting analysis. Anti-HA (HRP) and anti-Myc (HRP-conjugated, 2040S, CST) antibodies were used at 1:5,000-fold dilution.

### In vivo co-immunoprecipitation assays

The in vivo co-immunoprecipitation (Co-IP) assays were performed as previously described, with minor modifications^48^. In brief, 1 g of 6-day-old SD-grown F_1_ seedlings (*35* *S::PIF4-TAP* x *CDF2::3HA-CDF2*) was harvested at ZT-1. The seedlings were ground to fine powder in liquid nitrogen, semi-pure nuclei extractions were performed and nuclear proteins were released by a short sonication.

For co-immunoprecipitation, 30 μl of Dynabeads Protein G beads coated with 10 μl anti-Myc antibody was added to the diluted nuclear protein solution (0.5% Triton X-100, 1 mM EDTA, 20 mM Tris-HCl, pH 7.5, and 100 mM NaCl and 1× Protease Inhibitor Cocktail (Sigma-Aldrich)) and rotated for 45 min at 4 °C. The beads were washed five times with IP buffer. Proteins were eluted from the beads with 2× SDS–PAGE sample buffer and then subjected to immunoblotting analysis. For immunoblotting, anti-HA (HRP) and anti-Myc (HRP) antibodies were used at 1:2,500-fold dilution.

### Chromatin immunoprecipitation

ChIP methods were described previously with minor modifications^48^. For ChIP–seq of CDF2, 9 g above-ground tissue of 6-day-old SD-grown seedlings was harvested at ZT-1 and cross-linked for 10 min by vacuum filtration in phosphate-buffered saline solution containing 1% formaldehyde. For chromatin immunoprecipitation, 50 μl Dynabeads Protein G beads (Thermo Fisher Scientific) coated with 20 μl anti-HA antibody (ab9110, Abcam) was incubated for 4 h with 3 ml of the diluted chromatin solution (1% Triton X-100, 1 mM EDTA, 0.08% SDS, 15 mM Tris-HCl, pH 8.0, and 150 mM NaCl). After washing three times with wash buffer (1% NP-40, 1 mM EDTA, 0.1% SDS, 0.1% DOC (sodium deoxycholate, Sigma-Aldrich), 20 mM Tris-HCl, pH 8.0, and 150 mM NaCl), the immune complex was eluted from the beads in 400 μl elution buffer (1% SDS and 0.1 M NaHCO_3_). Next, samples were reverse cross-linked with 5 μl Proteinase K and 20 μl 5 M NaCl at 65 °C overnight and DNA was purified by a MinElute PCR Purification Kit (QIAGEN). Amounts of input DNA were quantified by fluorometry (Quantus, Promega) and the size of the fragments was analyzed by ultra-sensitive capillary electrophoresis (Agilent FEMTOpulse). ChIP–seq libraries were generated according to Ovation Ultralow Library Systems v2 (Tecan Genomics) with an adjusted cycle number that reflected the input amount. Sequencing-by-synthesis was performed on a HiSeq 3000 device at the Max Planck Genome-Centre Cologne in 150-bp single-read mode.

Raw single-end reads were preprocessed by removing potential sequencing adapters using cutadapt^49^ and trimming low quality bases at both ends with Trimmomatic^50^. The processed reads were mapped to the *Arabidopsis thaliana* genome version TAIR10 with Bowtie2 (ref. ^51^). Alignments with mapping quality less than 30 were discarded using SAMtools^52^. For initial peak calling using MACS v.2 (ref. ^53^), the resulting alignment files were fed in pairs consisting of ChIP–seq and corresponding input samples. For consistency with the differential binding assays, final merged peak calling was obtained with the DiffBind R package^54^. ChiPpeakAnno^55^ was used to assign peaks to genes if they were within 3 kb and 1 kb upstream or downstream from the transcription start or end site, respectively. The position of peaks relative to gene bodies was compared with a positional distribution obtained from 1,000 random peak sets with equal peak-size distributions as the observed set. Motif discovery was performed using MEME–ChIP^56^. CentriMo^57^ was used to determine the enrichment of motifs in the centre of peaks. Distances between consecutive motifs and the number of motifs per peak were obtained using custom python scripts. Differential binding assays of CDF2 peak locations between CDF2::HA-CDF2; *cdf2-1* and CDF2::HA-CDF2; *cdf2-1 pif4-2* were performed with DiffBind (Bioconductor, https://bioconductor.org/packages/release/bioc/html/DiffBind.html).

For ChIP–qPCR of RNA polymerase II, 9 g of above-ground tissue from 6-day-old SD-grown seedlings was harvested at ZT-1, cross-linked with 1 mM DSG (di(*N*-succinimidyl) glutarate, SYNCHEM) by vacuum filtration in phosphate-buffered saline solution for 10 min and then cross-linked for another 10 min with 1% formaldehyde. Chromatin immunoprecipitations were performed as described above with 30 μl Dynabeads Protein G beads coated with 10 μl anti-RNA polymerase II (ab5131, Abcam) and the resulting DNA was used for ChIP–qPCR. The primers used for ChIP–qPCR are listed in Supplementary Table 11.

### Gene expression and whole-transcriptomic RNA-sequencing analysis

To quantify *PIF4* and *CDF2* mRNA and protein accumulation in diurnal conditions, 6-day-old seedlings were harvested every 3 h and were flash-frozen in liquid nitrogen. Total RNA was extracted with an RNeasy plant Mini Kit (QIAGEN) with an on-column DNase (QIAGEN) treatment. cDNA was synthesized from 0.8 μg RNA using a QuantiTect Reverse Transcription Kit (QIAGEN). Real-time PCR was performed with iQ SYBR Green Supermix (Bio-rad) in a CFX384 Touch Real-Time PCR Detection System (Bio-rad). Two reference genes, *PP2A* and *APA1*, were used for normalization. Three technical replicates for each of three independent biological replicates were performed for each experiment and representative results are presented. The primers used for reverse transcription with quantitative PCR (RT–qPCR) are listed in Supplementary Table 1.

For RNA-seq, cotyledons and hypocotyls were dissected from 6-day-old seedlings at ~ZT-1–ZT-1.5 and were flash-frozen in liquid nitrogen. RNA was extracted as above and RNA quality was assessed by capillary electrophoresis (NanoChip, Agilent Bioanalyser). Poly-A RNA was enriched from 500 ng total RNA by the Poly(A) mRNA Magnetic Isolation Module (New England Biolabs). RNA-seq libraries were prepared using the Ultra II Directional RNA Library Prep Kit for Illumina (New England Biolabs). Thirteen cycles were applied to enrich library concentration. Sequencing-by-synthesis was performed on a HiSeq 3000 device at the Max Planck Genome-Centre Cologne in 2 × 150 bp paired-end read mode. Raw paired-end RNA-seq reads were cleaned using the same work-flow as for the ChIP–seq reads. The cleaned reads were used to quantify the expression levels of *Arabidopsis thaliana* transcripts in the AtRTD2^58^ and dataset using Salmon^59^. Differential expression analyses at the gene level were performed with the DESeq2 R package^60^. Principle component analysis of gene expression was performed with values of log_2_(FPKM + 1) expression level using the prcomp function R. All Gene Ontology-term enrichment analyses related to the ChIP–seq and RNA-seq datasets were performed using the TopGO R package^61^. All statistical tests related to the NGS (next-generation sequencing) data were performed in R.

### Protein expression and purification

Codons of the coding sequences of CDF2^DOF^ and PIF4^bHLH^ domains from *Arabidopsis* *thaliana* were optimized to *E*. *coli* and cloned into pMAL-c5X-His Vector (NEB). CDF2^DOF^ domain was between 133 and 201 aa, whereas the PIF4^bHLH^ domain was between 248 and 317 aa. The wild-type CDF2^DOF^, PIF4^bHLH^ and mutant proteins were induced by 0.7 mM IPTG (Sigma) and expressed in ArcticExpress cells (Agilgent Technologies) at 12 °C, overnight. The *E*. *coli* cells were collected by centrifugation, resuspended in wash buffer (25 mM Bis-Tris pH 8.0, 150 mM NaCl and 15 mM imidazole) and sonicated to prepare cell lysates. The proteins were purified using Ni-NTA beads (GE), the bound proteins were washed five times with wash buffer and eluted using elution buffer (25 mM Bis-Tris pH 8.0, 150 mM NaCl and 250 mM imidazole). The eluted proteins were further purified by size-exclusion chromatography (HiLoad 200, GE Healthcare) in buffer containing 25 mM Bis-Tris pH 8.0, and 150 mM NaCl.

### Structural modelling

The structures of CDF2 and PIF4 full length, and CDF2 DOF domain and PIF4 bHLH domain were predicted using AlphaFold^35^ and I-TASSER Suite^36^, respectively. The modelled structure of CDF2 DOF–DNA complex was based on the zif268–DNA complex (PDB ID 1ZAA)^37^. The dimer and tetramer modelled structures of PIF4 bHLH were based on MYC2 bHLH–DNA complex with G-box (PDB ID 5GNJ)^39^. The structure data were processed using the program Coot and PyMOL softwares.

### Gel-shift assay (EMSA)

The long double-stranded DNA probe (95 bp) covering the two G-boxes and two DOF-binding sites was synthesized by PCR using 5′-Cy5-labelled oligo primers. the short double-stranded DNA probe (38 bp) covering one G-box and one DOF-binding site was synthesized by annealing single-stranded 5′-Cy5-labelled oligo in annealing buffer (10 mM Tris (pH 8.0), 50 mM NaCl, and 1 mM EDTA (pH 8.0)). Binding reactions were carried out in buffer containing 10 mM Tris, 50 ng μl^−1^ Poly (dI-dC), 50 mM KCl, 10 mM KCl, 1 mM DTT, 5% glycerol and 0.1% NP-40. Samples were kept in the light on ice for 30 min and were then loaded onto 6% DNA Retardation Gels (Thermo Fisher Scientific) and run in 0.5× Tris/Borate/EDTA buffer at room temperature for 90 min at 70 V. Binding signals were visualized using a ChemiDoc MP Imaging System (Bio-Rad). The primers used for DNA probes are listed in Supplementary Table 11.

### Reporting summary

Further information on research design is available in the Nature Research Reporting Summary linked to this article.

## Supplementary information

Supplementary Information Supplementary Tables 1–11: 1, CDF2 ChIP–seq binding peaks assigned to neighbouring genes; 2, PIF4 ChIP–seq binding peaks assigned to neighbouring genes; 3, Common binding peaks of CDF2 and PIF4 assigned to neighbouring genes; 4, ChIP–seq differential binding of CDF2 in *pif4* assigned to neighbouring genes; 5, Common genes assigned by differential binding peaks of CDF2 in *pif4* and PIF4-binding peaks; 6, Differentially expressed genes (DEGs) in cotyledons and hypocotyls of *pif4*; 7, Differentially expressed genes (DEGs) in cotyledons and hypocotyls of *cdfq*; 8, Co-regulated genes in cotyledons of *pif4* and *cdfq*; 9, Co-regulated genes in hypocotyls of *pif4* and *cdfq*; 10, Common DEGs that were bound by both PIF4 and CDF2, and were bound less strongly by CDF2 in *pif4*; 11, Primers used in this study.

Reporting Summary

## Data Availability

All data needed to evaluate the conclusions in the paper are present in the paper or the supplementary materials. Mutants, transgenic plants and all plasmid constructions using CDF2 and PIF4 are available from G.C. under a material transfer agreement with the Max Planck Institute for Plant Breeding Research. Raw data are available from RNA-seq series PRJNA747146 and ChIP–seq series PRJNA747820. Source data are provided with this paper.

## Source data

Source Data Fig. 1 Statistical source data.
Source Data Fig. 2 Unprocessed western blots and gels.
Source Data Fig. 4 Statistical source data.
Source Data Fig. 5 Unprocessed western blots and gels.
Source Data Extended Data Fig. 1 Statistical source data.
Source Data Extended Data Fig. 2 Statistical source data.
Source Data Extended Data Fig. 2 Unprocessed western blots and gels.
Source Data Extended Data Fig. 7 Unprocessed western blots and gels.
