# Supplementary material for: PIF4 enhances DNA binding of CDF2 to co-regulate target gene expression and promote Arabidopsis hypocotyl cell elongation
Source: Nat Plants. 2022 Aug 15;8(9):1082–93. doi: 10.1038/s41477-022-01213-y (PMC9477738; doi:10.1038/s41477-022-01213-y)
Supplement: Supplementary file 2 — Reporting Summary [file 41477_2022_1213_MOESM2_ESM.pdf]

nature portfolio

Corresponding author(s): George Coupland

Last updated by author(s): June 10th, 2022

Reporting Summary

Nature Portfolio wishes to improve the reproducibility of the work that we publish. This form provides structure for consistency and transparency in reporting. For further information on Nature Portfolio policies, see our Editorial Policies and the Editorial Policy Checklist.

Statistics

For all statistical analyses, confirm that the following items are present in the figure legend, table legend, main text, or Methods section.

n/a	Confirmed	
☐	☒	The exact sample size (*n*) for each experimental group/condition, given as a discrete number and unit of measurement
☐	☒	A statement on whether measurements were taken from distinct samples or whether the same sample was measured repeatedly
☐	☒	The statistical test(s) used AND whether they are one- or two-sided<br>*Only common tests should be described solely by name; describe more complex techniques in the Methods section.*
☐	☒	A description of all covariates tested
☐	☒	A description of any assumptions or corrections, such as tests of normality and adjustment for multiple comparisons
☐	☒	A full description of the statistical parameters including central tendency (e.g. means) or other basic estimates (e.g. regression coefficient) AND variation (e.g. standard deviation) or associated estimates of uncertainty (e.g. confidence intervals)
☐	☒	For null hypothesis testing, the test statistic (e.g. *F*, *t*, *r*) with confidence intervals, effect sizes, degrees of freedom and *P* value noted<br>*Give P values as exact values whenever suitable.*
☐	☒	For Bayesian analysis, information on the choice of priors and Markov chain Monte Carlo settings
☐	☒	For hierarchical and complex designs, identification of the appropriate level for tests and full reporting of outcomes
☐	☒	Estimates of effect sizes (e.g. Cohen's *d*, Pearson's *r*), indicating how they were calculated

Our web collection on statistics for biologists contains articles on many of the points above.

Software and code

Policy information about availability of computer code

Data collection	MorphoGraphX;<br>R;<br>AlphaFold Protein Structure Database (https://alphafold.ebi.ac.uk/);<br>Protein Data Bank (PDB, https://www.rcsb.org/)<br>Pymol (https://pymol.org/2/)
Data analysis	Image J; MorphoGraphX and R.

For manuscripts utilizing custom algorithms or software that are central to the research but not yet described in published literature, software must be made available to editors and reviewers. We strongly encourage code deposition in a community repository (e.g. GitHub). See the Nature Portfolio guidelines for submitting code & software for further information.

Data

Policy information about availability of data

All manuscripts must include a data availability statement. This statement should provide the following information, where applicable:

- Accession codes, unique identifiers, or web links for publicly available datasets
- A description of any restrictions on data availability
- For clinical datasets or third party data, please ensure that the statement adheres to our policy

Raw data for RNA-seq and ChIP-seq will be released on 08. 2022.
Raw data are for RNA-seq series RNA-seq series PRJNA747146 and ChIP-seq series PRJNA747820.

Field-specific reporting

Please select the one below that is the best fit for your research. If you are not sure, read the appropriate sections before making your selection.

☒ Life sciences ☐ Behavioural & social sciences ☐ Ecological, evolutionary & environmental sciences

For a reference copy of the document with all sections, see nature.com/documents/nr-reporting-summary-flat.pdf

Life sciences study design

All studies must disclose on these points even when the disclosure is negative.

Sample size

Analysis of Arabidopsis hypocotyl length. Statistical analysis was performed via Tukey's least significant difference (LSD) test ($P \leq 0.05$). n = 28 hypocotyls.

Analysis of epidermal cells in non-dividing cell files of Arabidopsis hypocotyls. Significant differences among genotypes were observed ($p < 0.05$, using ANOVA followed by Tukey's pairwise multiple comparison); n = 4 hypocotyls.

Data exclusions

No data were excluded during the analysis.

Replication

At least three independent experiments were done to verify the reproducibility of the findings. And all the replicates were successful.

Randomization

Seedlings were grown randomly in growth chamber and seedlings were harvested randomly for data collection.

Blinding

The investigators were blinded to group allocation during data colection and/or analysis.

Reporting for specific materials, systems and methods

We require information from authors about some types of materials, experimental systems and methods used in many studies. Here, indicate whether each material, system or method listed is relevant to your study. If you are not sure if a list item applies to your research, read the appropriate section before selecting a response.

Materials & experimental systems

n/a	Involved in the study	
☐	☒	Antibodies
☒	☐	Eukaryotic cell lines
☒	☐	Palaeontology and archaeology
☒	☐	Animals and other organisms
☒	☐	Human research participants
☒	☐	Clinical data
☒	☐	Dual use research of concern

Methods

n/a	Involved in the study	
☐	☒	ChIP-seq
☒	☐	Flow cytometry
☒	☐	MRI-based neuroimaging

Antibodies

Antibodies used

anti-HA antibody (ab9110, Abcam); anti-Myc antibody (2276S, Cell Signaling Technology); anti-HA antibody (12013819001, Roche) or anti-Actin antibody (sc-47778, Santa Cruz); anti-Myc antibody (HRP-conjected, 2040S, Cell Signaling Technology).

Validation

Anti-HA (HRP) and anti-Myc (HRP-conjected, 2040S, CST) antibodies were used at 1:5000-fold dilution for western blot for in vitro pull down experiments.
Anti-HA (HRP) and anti-ACT (HRP) antibodies were used at 1:2,000 and 1:4,000-fold dilutions for western blot for diurnal protein accumulation detection.

ChIP-seq

Data deposition

☒ Confirm that both raw and final processed data have been deposited in a public database such as GEO.

☒ Confirm that you have deposited or provided access to graph files (e.g. BED files) for the called peaks.

Data access links
May remain private before publication.

Access for the referees is provided to all the raw peak files through the following links
CDF2::HA-CDF2_cdf2_pif4_rep-1.narrowPeak http://84.22.105.30/natureplantshegao/CDF2::HA-CDF2_cdf2_pif4_rep-1.narrowPeak

CDF2::HA-CDF2_cdf2_pif4_rep-2.narrowPeak http://84.22.105.30/natureplantshegao/CDF2::HA-CDF2_cdf2_pif4_rep-2.narrowPeak
CDF2::HA-CDF2_cdf2_pif4_rep-3.narrowPeak http://84.22.105.30/natureplantshegao/CDF2::HA-CDF2_cdf2_pif4_rep-3.narrowPeak
CDF2::HA-CDF2_cdf2_rep-1.narrowPeak http://84.22.105.30/natureplantshegao/CDF2::HA-CDF2_cdf2_rep-1.narrowPeak
CDF2::HA-CDF2_cdf2_rep-2.narrowPeak http://84.22.105.30/natureplantshegao/CDF2::HA-CDF2_cdf2_rep-2.narrowPeak
CDF2::HA-CDF2_cdf2_rep-3.narrowPeak http://84.22.105.30/natureplantshegao/CDF2::HA-CDF2_cdf2_rep-3.narrowPeak

Files in database submission

The files that will be in the database submission correspond to the MACS2 peak calling files. They are named according to the Chip sample
CDF2::HA-CDF2_cdf2_pif4_rep-1.narrowPeak
CDF2::HA-CDF2_cdf2_pif4_rep-3.narrowPeak
CDF2::HA-CDF2_cdf2_rep-2.narrowPeak
CDF2::HA-CDF2_cdf2_pif4_rep-2.narrowPeak
CDF2::HA-CDF2_cdf2_rep-1.narrowPeak
CDF2::HA-CDF2_cdf2_rep-3.narrowPeak

Genome browser session
(e.g. UCSC)

a current session is:
http://84.22.105.30/natureplantshegao/?session=share-9QD2enlGV6&password=3cURj

alternatively the following link can be used and the linear genome view will also lead to the same browser data
http://84.22.105.30/natureplantshegao

Methodology

Replicates

ChIP-seq experiments were done with three independent biological repeats.

Sequencing depth

Sample	TotalReads	UniquelyMapped	MaxReadLength	layout
CDF2::HA-CDF2_cdf2_rep-1	25469658	18530900	151	single
CDF2::HA-CDF2_input_rep-1	22914897	16662023	151	single
CDF2::HA-CDF2_cdf2_rep-2	24122923	18232832	151	single
CDF2::HA-CDF2_input_rep-2	22315072	17087284	151	single
CDF2::HA-CDF2_cdf2_rep-3	24150078	17576376	151	single
CDF2::HA-CDF2_input_rep-3	21005583	15278099	151	single
CDF2::HA-CDF2_cdf2_pif4_rep-1	26571112	20705627	151	single
CDF2::HA-CDF2_input_pif4_rep-1	23928821	19346629	151	single
CDF2::HA-CDF2_cdf2_pif4_rep-2	27397745	21268854	151	single
CDF2::HA-CDF2_input_pif4_rep-2	27132603	21539254	151	single
CDF2::HA-CDF2_cdf2_pif4_rep-3	22604576	17600545	151	single
CDF2::HA-CDF2_input_pif4_rep-3	27377627	21983733	151	single

Antibodies

anti-HA antibody (ab9110, Abcam).

Peak calling parameters

Bowtie2 Genome index for Arabidopsis thaliana was constructed using bowtie2-build [Athaliana.fasta] [genome] (rest of parameters was left to their default values). Reads were mapped using the following command: bowtie2 -p 4 -x genome -U [sample-fastq-file] | samtools view -Shb > [sample.bam]. Resulting bam files were read-position-based sorted using samtools sort -o [sorted.bam] [sample.bam]. Finally reads with mapping quality lower than 30 were removed by executing: samtools view -q 30 -h -b [sorted.bam] > [final.bam]. macs2 callpeak -t [ChIP.bam] -c [input.bam] -m 2 20 -q 0.01 -n [output] -g 120e6 -B --SPMR. Finally diffbind was used and only merged peaks were considered that were present in all three replicates.

Data quality

As described in the Peak calling parameters. Stringent removal of alignments of mapping quality < 30 increased the probability that the reads were uniquely mapped. Next, due to the large number of initial peaks we used FDR cutoff of 0.01. Finally, further stringent filtering was performed by requiring a peak to be observed in all three replicates. The following summary was generated for the peak sets

Chip	totalPeaks	enriched5plus
CDF2::HA-CDF2_cdf2_rep-1	15102	2800
CDF2::HA-CDF2_cdf2_rep-2	14361	2771
CDF2::HA-CDF2_cdf2_rep-3	11542	1401
CDF2::HA-CDF2_cdf2_pif4_rep-1	7306	284
CDF2::HA-CDF2_cdf2_pif4_rep-2	13275	2165
CDF2::HA-CDF2_cdf2_pif4_rep-3	10655	1927

Software

Raw peaks were called for each ChIP-seq,Input sample pair using the MACS2 software. Given that one of our main goals in the study was to determine differential binding, raw peaks were further processed using the diffbind R package.
