# Supplementary figures and images for: PIF4 enhances DNA binding of CDF2 to co-regulate target gene expression and promote Arabidopsis hypocotyl cell elongation
Source: Nat Plants. 2022 Aug 15;8(9):1082–93. doi: 10.1038/s41477-022-01213-y (PMC9477738; doi:10.1038/s41477-022-01213-y)

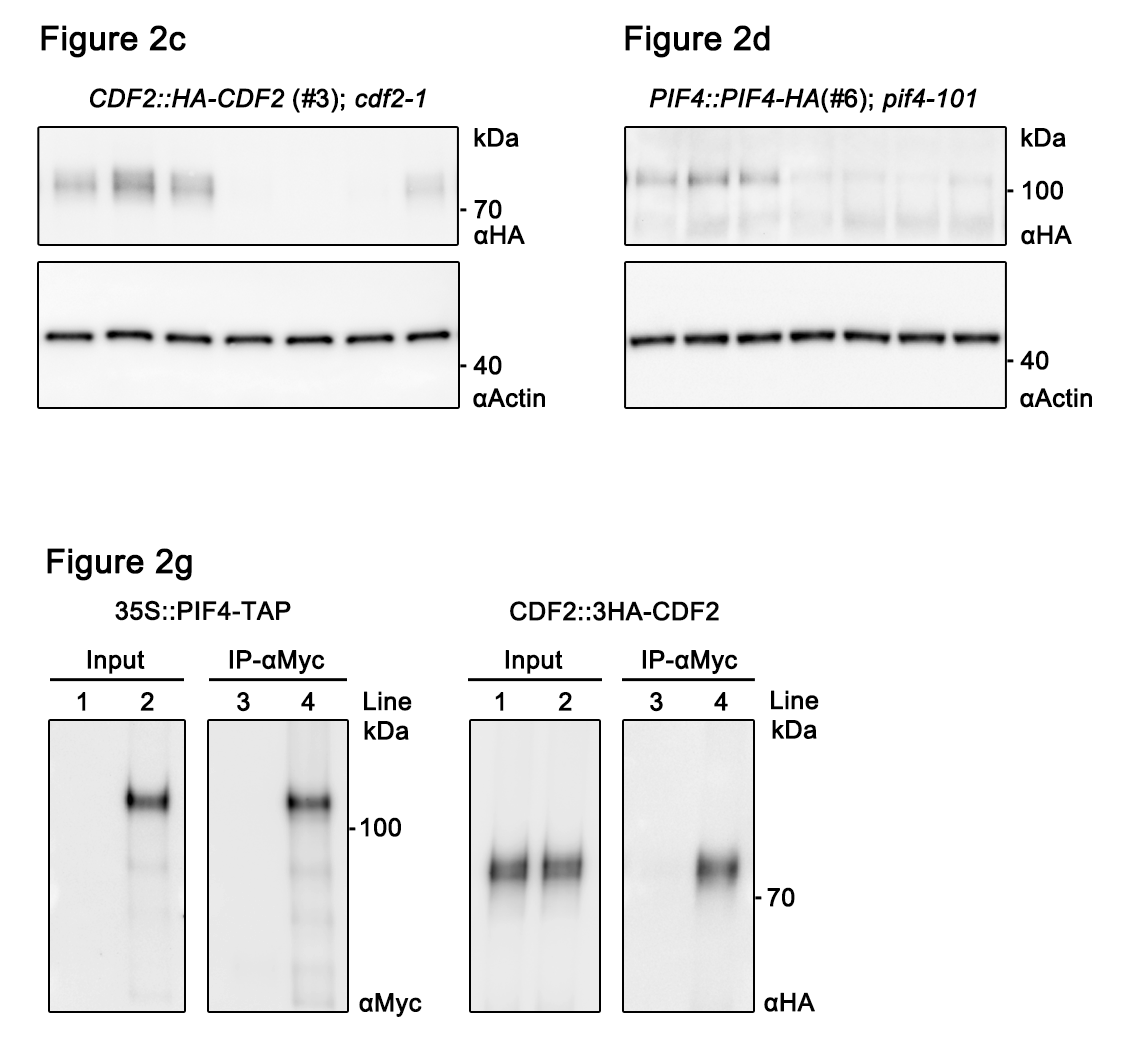

Supplement: Source Data Fig. 2 — Unprocessed western blots and gels. [file 41477_2022_1213_MOESM4_ESM.tif]

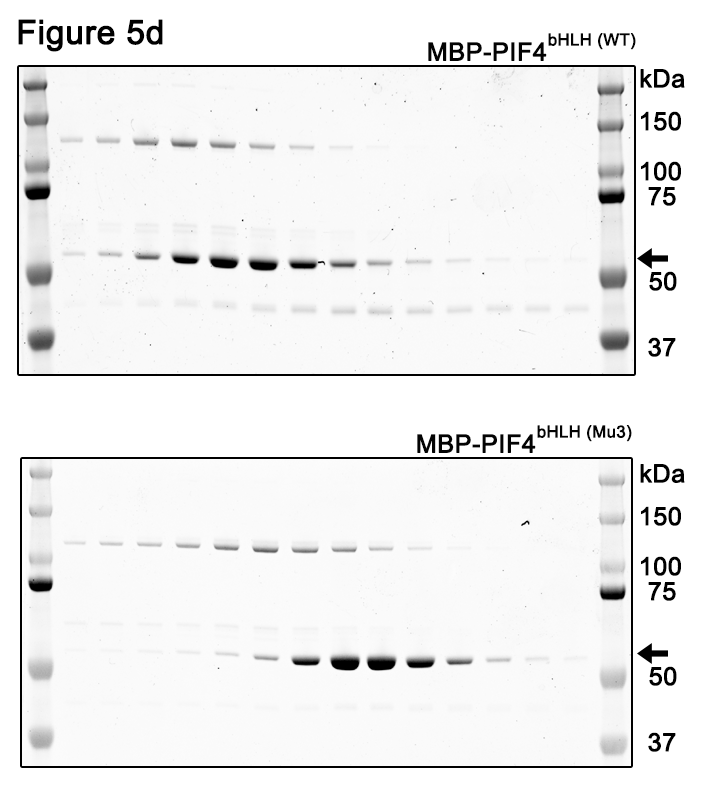

Supplement: Source Data Fig. 5 — Unprocessed western blots and gels. [file 41477_2022_1213_MOESM6_ESM.tif]

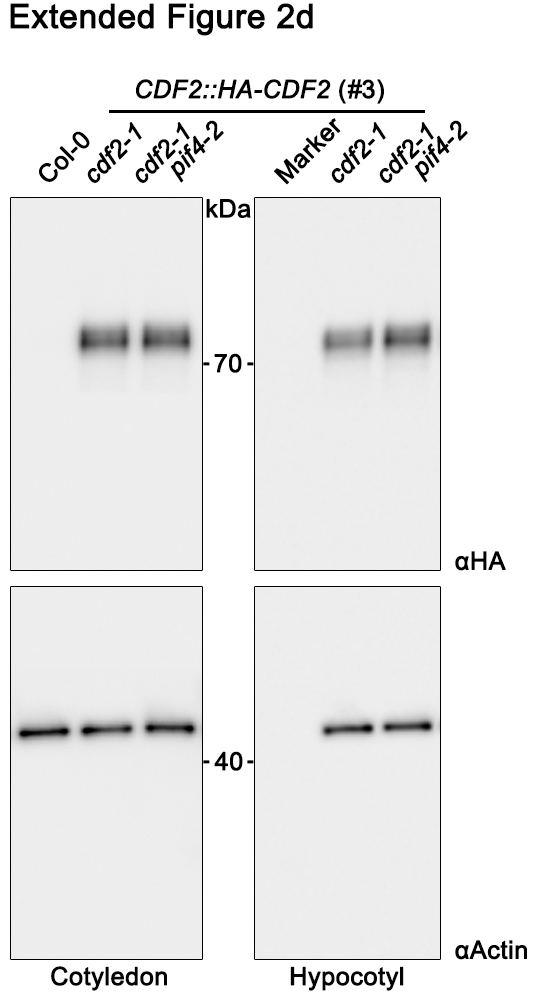

Supplement: Source Data Extended Data Fig. 2 — Unprocessed western blots and gels. [file 41477_2022_1213_MOESM9_ESM.tif]

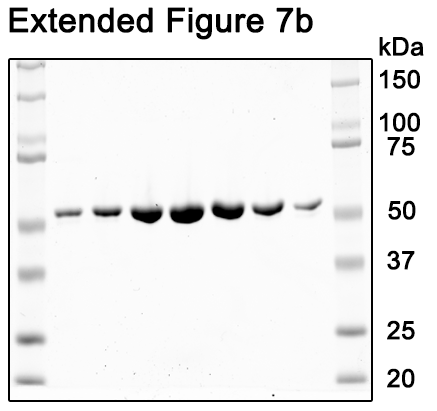

Supplement: Source Data Extended Data Fig. 7 — Unprocessed western blots and gels. [file 41477_2022_1213_MOESM10_ESM.tif]
